# Optimizing hyper-parameters of neural networks with swarm intelligence: A novel framework for credit scoring

**DOI:** 10.1371/journal.pone.0234254

**Published:** 2020-06-05

**Authors:** Runchi Zhang, Zhiyi Qiu

**Affiliations:** 1 Postdoctoral Work Station of Bank of Jiangsu Co., Ltd, Nanjing, Jiangsu, China; 2 Postdoctoral Research Station of Nanjing University, Nanjing, Jiangsu, China; 3 School of Public Economics and Administration, Shanghai University of Finance and Economics, Shanghai, China; Newcastle University, UNITED KINGDOM

## Abstract

Neural networks are widely used in automatic credit scoring systems with high accuracy and outstanding efficiency. However, in the absence of prior knowledge, it is difficult to determine the set of hyper-parameters, which makes its application limited in practice. This paper presents a novel framework of credit-scoring model based on neural networks trained by the optimal swarm intelligence (SI) algorithm. This framework incorporates three procedures. Step 1, pre-processing, including imputation, normalization, and re-ordering of the samples. Step 2, training, where SI algorithms optimize hyper-parameters of back-propagation artificial neural networks (BP-ANN) with the area under curve (AUC) as the evaluation function. Step 3, test, applying the optimized model in Step 2 to predict new samples. The results show that the framework proposed in this paper searches the hyper-parameter space efficiently and finds the optimal set of hyper parameters with appropriate time complexity, which enhances the fitting and generalization ability of BP-ANN. Compared with existing credit-scoring models, the model in this paper predicts with a higher accuracy. Additionally, the model enjoys a greater robustness, for the difference of performance between training and testing phases.

## Introduction

Credit scoring refers to the process using statistics to classify applicants for credit into different risk categories [1], in order to “determine the likelihood that a prospective borrower will default on a loan” [2]. The history of credit scoring is relatively short as about sixty years [3], despite the long history of credit which could be traced back to 2000 BC [4]. In practice, the credit scoring transforms “relevant data into numerical measures that guide credit decisions” [5]. Therefore, a variety of statistical models are applied in the process. The simple parametric statistical model, linear discriminate analysis (LDA) is one of the first models for credit scoring, although it is questioned because of the presumed normal distribution of data [6]. This deficiency of LDA is largely overcome by some sophisticated models like logistic regression, k nearest neighbor [7, 8], decision trees [8, 9], and neural networks [8, 10–13]. Notably, large financial intermediaries like American Express and Security Pacific Bank (SPB) build their credit scoring system on the basis of neural network, for this model outperforms others by 10% more accuracy [14]. A body of prior literature focusing on the techniques of credit scoring [15–20] are mostly based on classical statistic theories, which are less adaptive in the context of large sample.

Fintech as the fusion of finance and technology [21] is applied to credit scoring recently. However, the performance of these approaches relies on the parameters and the application is limited because of the difficult in determining parameter with lack of prior knowledge. Zhao, Xu [22] tests that fintech approaches like the neural network perform well as long as the parameters are properly set. In other word, parameter setting determines the performance of these approaches. Notably, some remarkable progress takes place in swarm intelligence algorithm [23, 24]. Based on the logic of natural selection, the approaches of swarm intelligence algorithm mimics individual and in-group behaviors of species to seek the optimal solution. As Hurley and Adebayo [25] suggests, “all data is credit data”. This paper attempts at a novel framework combined with conventional credit information in credit-scoring industry with the emergence of big data technology.

Notably, some state-of-the-art techniques are proposed to determine neural network architecture in recent decades [26–28]. However, these techniques, consuming several GPU-days, are more applicable with the scenario of image/audio recognition where high-dimension large-size datasets prevail. To the contrary, the credit scoring is a completely different scenario. First, the datasets of credit is smaller sized with less dimensions and consequently the above-mentioned techniques are prone to overfit. Second, high-performance computing (HPC) is inaccessible to most banking practitioners, especially small-/medium-size depository institutions. Thus, the purpose of this research is to construct a realistic framework tailored for credit scoring to optimize the hyper-parameters of neural network with swarm intelligence algorithm. This paper further benchmarks the performance of the novel framework against classical as well as hybrid or ensemble models proposed in recent literature [29–34]. This paper is to answer the following questions. First, does the neural network with hyper-parameters determined by swarm intelligence algorithm outperform the classical credit-scoring models (i.e. logistic regression, naive Bayesian, discriminant analysis, k nearest neighbor, decision tree, support-vector machine, K-means, and random forest) and state-of-the-art models proposed in recent literature [29–34]? Second, are the fitting and generalization ability of a neural network steady after its parameters determined by swarm intelligence algorithms? Third, does our framework perform robustly with increasing hidden layers of neural network? Fourth, what is the comparative advantage of our framework against the state-of-the-art techniques for optimizing neural networks [26–28]?

This paper sheds a new light on the application of swarm intelligence algorithm to the credit scoring area. To address this purpose, this paper proposes a novel credit-scoring framework to determine the optimal SI algorithm for hyper-parameter optimization of neural network and carries out an experiment to test the generalization and robustness of neural network trained by swarm intelligence algorithm. Specifically, eight other prevalent credit-scoring models as well as some hybrid or ensemble models constructed in recent literature [29–34] make up the control group and seven swarm intelligence algorithms are extracted from prior literature. The neural network with parameters trained by the seven swarm intelligence algorithms are included in the treated group. This paper compares the performance of models in the treated and control groups to classify the appropriate model for credit scoring. The findings shows that models constructed within this framework outperforms models in the control group. The application of fintech in this paper implies that, despite the challenges brought by fintech [21], tech-driven services are complements rather than replacement of the traditional banking system [35, 36] and commercial banks would embrace with fintech to gain a new growth [35, 37].

The rest of this paper is organized as follows. Section 2 constructs a theoretical framework of the prevalent classical credit scoring models and the typical swarm intelligence algorithm approaches. In Section 3, a novel framework is proposed for optimizing hyper-parameter of neural networks with swarm intelligence algorithms. Section 4 describes the data used in the empirical research and findings are reported and analyzed in Section 5. In the last section, this paper draws conclusion and provides suggestions on model selection of credit scoring accordingly.

## Theories of algorithm

### Prevalent classical models for credit scoring

Credit scoring models support lenders during the decision-making process of loans. A body of credit scoring models developed into maturity in the recent decades, including statistics-based models like logistic regression, naive Bayes, determinant analysis [38, 39] and machine learning based models like K nearest neighbor, decision tree, support vector machine, artificial neural network [40–42]. As is mentioned in the section of introduction, artificial neural network is widely accepted for its outstanding accuracy and is selected as the underlying model of this paper. These models are applies to different scenarios because of distinct assumptions and instance characteristics.

#### Artificial neural network (ANN)

The artificial neural network is an important quantitative technique in credit scoring [43], which is widely used in the context of microfinance [44], imbalanced data [45], real-time assessment [39], etc. The technique of neural network model has evolved into different forms to deal with the credit scoring problems; e.g. partial logistic artificial neural network [46], artificial metaplasticity neural network [47], and hybrid neural networks [48]. Prior experiments show that the neural networks outperforms a bunch of conventional techniques (e.g. discriminant analysis, probit analysis, logistic regression, etc.) in credit scoring [49–51]. Furthermore, the neural networks trained by more sophisticated algorithms outperform those trained by ordinary gradient descent [22, 52]. Besides, the hybrid of neural network and genetic algorithm proves as excellent classifier in credit scoring [53]. However, some issues remain in the application of neural network to credit scoring. For example, the determination of training-to-validation sample ratio remains controversial in the prior literature [54, 55]. Notably, similar to the most of machine learning techniques, the neural network is prone to overfitting and consequently poor generalization [56]. Thus, the aim of this paper is to improve the generalization of neural network with swarm intelligence algorithms.

The approach of artificial neural network (ANN) stimulates the neural network of human brain [57]. With artificial neuron as the unit of information operation, the weight value of connection between artificial neurons indicates the intensity of connection. The connection and the structure reflect how the information is represented, transmitted, and operated in the network. Back propagation (BP) is the most prevalent neural network in the context of credit scoring. We apply recurrent back-propagation in this paper, which is fed forward until a fixed value is achieved while the error is computed and propagated backward. A typical neural network consists of input layer, hidden layer, and output layer. We introduce the training procedures as follows. First, the network is fed forward: hidden layer accepts data from input layer and modifies them with non-linear transformation before output. Once the output value is generated, we measure the difference between actual and desired output value and obtain the error value. In this stage, the error value is transferred backward from output layer to hidden layer and then to input layer. At the same time, the error value is shared across layers and the weight value of every unit is adjusted accordingly. Intended for the gradient decrease of the error value, we adjust the connection between layers (i.e. input-hidden connection and hidden-output connection) and the threshold. This training process goes on until we classify the network parameters (i.e. weight and threshold values) applicable to the minimized error value. After the above-mentioned training process, when fed with an input value, the neural network automatically outputs values with minimal error after non-linear transformation.

#### Logistic regression

Logistic regression proposed by Berkson [58] is most widely used in both industry and academy of banking thanks to its simple architecture and time complexity [59–61].

Conditional probability for logistic regression is given by

$$P(y_{i}=1|x_{i})=\frac{e^{\left(\beta_{0}+\beta_{1}x_{i1}+\beta_{2}x_{i2}+\cdots +\beta_{m}x_{im}\right)}}{1+e^{\left(\beta_{0}+\beta_{1}x_{i1}+\beta_{2}x_{i2}+\cdots +\beta_{m}x_{im}\right)}}$$ (1)

$$P(y_{i}=0|x_{i})=\frac{1}{1+e^{\left(\beta_{0}+\beta_{1}x_{i1}+\beta_{2}x_{i2}+\cdots +\beta_{m}x_{im}\right)}}$$ (2)

where *β*_0_, *β*_1_, ⋯, *β*_*m*_ are estimated with maximum likelihood estimation (MLE). To be specific, as the independent variable *y*_*i*_ takes value either zero or one, then

$$P(y_{i})=P_{i}^{y_{i}}{\left(1-P_{i}\right)}^{1-y_{i}}$$ (3)

Since the instances are independent from each other, the likelihood function is given by

$$L(\theta )=\prod_{i=1}^{n}P_{i}^{y_{i}}{\left(1-P_{i}\right)}^{1-y_{i}}$$ (4)

and the logarithmic function is

$$\begin{array}{l} \text{ln}\;L(\theta )=\text{ln}(\prod_{i=1}^{n}P_{i}^{y_{i}}{\left(1-P_{i}\right)}^{1-y_{i}})=\sum_{i=1}^{n}\left[y_{i}\;\text{ln}(P_{i})+(1-y_{i})\;\text{ln}(1-P_{i})\right]\\ =\sum_{i=1}^{n}\left[y_{i}\;\text{ln}(\frac{P_{i}}{1-P_{i}})+\text{ln}(1-P_{i})\right] \end{array}$$ (5)

The value of *β*_0_, *β*_1_, ⋯, *β*_*m*_ is estimated when the partial derivatives with respect to the four variables equal zero. However, instead of a closed-form solution, we estimate the non-linear likelihood function with iteration. As is suggested in prior literature, the logistic regression performs weakly when solving non-linear problems [62].

#### Naive Bayesian (NB) approach

The approach of Naive Bayes (NB) is born from the classical Bayesian approach of statistics and provides theoretical justification for classifiers that even do not use Bayesian theorem explicitly [62]. Based on the solid theoretical framework of statistics, the NB model remains robust in the case of missing value. However, the underlying assumption that all the indicators are independent from each other is too strong for the real world. The Bayesian conditional probability of event y and x is given by

$$p(y|x)=\frac{p(y,x)}{p(x)}=\frac{p(x|y)\times p(y)}{p(x)}$$ (6)

i.e. diving the probability that both event y and x take place by the probability that event x takes place measures the probability of event x with the condition that event y takes place. In the context of credit scoring, event x refers to the case that a certain character (measured by an indicator) of the instance takes a particular value. Specifically, instance of credit history is divided into several categories (e.g. two categories: *y*_*i*_ = 1 for default; *y*_*i*_ = 0 for non-default) and *p*(*y*) in Eq (6) refers to the frequency of each category. Then *p*(*x* | *y*) in Eq (6) equals the frequency of indicators in the subsample of each category and *p*(*x*) equals the percentage of indications in the full sample. Thereby, *p*(*y* | *x*) is measured according to Eq (6).

If there are more than one indicator for the independent variable, then

$$\begin{array}{l} p(y_{j}|x_{i1},x_{i2},\cdots ,x_{im})=\frac{p(y_{j})p(x_{i1},x_{i2},\cdots ,x_{im}|y_{j})}{p(x_{i1},x_{i2},\cdots ,x_{im})}=\\ \alpha \times p(y_{j})\times p(x_{i1},x_{i2},\cdots ,x_{im}|y_{j})=\alpha \times p(y_{j})\times \overset{m}{\underset{k=1}{∐}}p(x_{ik}|y_{j}) \end{array}$$ (7)

#### Discriminant analysis (DA)

Discriminant analysis (DA) proposed by [63] is often cited to compare with other techniques in credit scoring [64, 65]. This approach forms classification criteria based on the instance with categories known and predict the unknown categories according to such criteria. The DA is either parametric or non-parametric. The parametric DA constructs the model with a certain assumption of instance distribution (e.g. normal distribution). However, the model is constructed biased because of the unobservable distribution so that parametric DA is not widely used and DA performs weakly when dealing with non-linear problems [62]. For instead, the non-parametric DA prevails in the context of credit scoring. This approach investigate instance distribution with non-parametric method and construct classification criteria accordingly. Thus, results of non-parametric DA are more robust.

#### K nearest neighbor (KNN)

As one of the most classical method of data mining, k nearest neighbor (KNN) is carried out as follows. Suppose *x*_*i*_ remains to categorize. In the instance set whose category is known, find k instances most similar to (nearest to) *x*_*i*_, known as k nearest neighbors of *x*_*i*_. According to the rule of majority voting, *x*_*i*_ is classified into the neighbor’s category with the largest number of instances. If *k* = 1, then *x*_*i*_ is classified into the same category as its nearest neighbor. Since the KNN relies on the comparison with a set containing known values rather than estimation, this approach is efficient in terms of modelling. However, the predictive accuracy of KNN is determined by the measure of distance and the cardinality *k* of the neighborhood [62].

#### Decision tree (DT)

Decision tree (DT), a basic technique of ensemble learning [66], is another prevalent machine-learning-based approach to credit scoring [67]. With some new techniques introduced [68, 69], the DT approach is efficient in categorization and shows results in an explicit manner for interpretation. However, this approach leads to biased results when the instance is time series with complex categories. In the framework of greedy algorithm, the DT construct a tree-shape structure. First, classify the optimal value of a certain indicator and classify the instance accordingly. Then, divide each subsample with optimal value until the predefined stopping criterion reached. To be specific,

Step 1: take the full sample set as the root node of the tree.
Step 2: test every possible classification of every indicator until the optimal value identified in the recursion.
Step 3: set decision nodes using the optimal value in Step 2 and classify the root node into leaf nodes.
Step 4: repeat Step 2 and 3 until every leaf node is pure enough.

As core of the DT approach, purity measures the ratio of homogenous instances in a leaf node over the full sample; i.e. one leaf node is “purer” as such ratio is higher. The optimal classification refers to the one that improve the purity most in the recursion. The concept of entropy shown in Eq (8) measures the uncertainty of categorization in each subsample.

$$Info(D)=-\sum_{i=1}^{c}p_{i}\;{\text{log}}_{2}(p_{i})$$ (8)

where *c* denotes the number of categories in the instance set *D* (e.g. in real-world credit scoring, *c* equals two as the instance set is categorized into “default” and “non-default”). *p*_*i*_ denotes the ratio of subsample size in category *i* over the full sample set *D*; i.e. $p_{i}=\frac{\left|D_{i}\right|}{\left|D\right|}$. On general, a large value of entropy indicates that an increasing body of information is required for categorization.

#### Support vector machine (SVM)

The support vector machine (SVM) is employed as a technique of credit scoring in the past decade [19, 70–72]. Prior literature using real-world credit scoring data from the US and Taiwan (China) indicates that support vector machines achieves accuracy comparable of that of neural networks [73].

The SVM is a non-probabilistic binary linear classifier, classifying the optimal hyperplane to split instance in the space to the maximum [74]. The term “to the maximum” indicates that the distance between subsample and the hyperplane is maximized and the error in categorization is minimized thereby. Applying kernel function, the SVM simplifies classification applicable to various scenarios. However, its performance relies on the selection of kernel function and it consumes a large storage for computation.

Three types of kernel function are used to classify the optimal hyperplane: linear, polynomial, and radial basis function. The linear kernel function divides instances by a plane and attempts to classify the hyperplane in the original feature space. The polynomial kernel function transforms the original instances into high-dimensional instances with polynomial characteristics and then divides these transformed instances with a curve. The radial basis function (RBF) classifies the hyperplane after mapping instances into higher dimensional feature space via the RBF. In most cases, the RBF outperforms the other two kernel functions. In this paper, all these three kernel functions and their parameter set are employed to obtain the best performance via grid search method.

#### K-means

The K-means clustering method is an unsupervised learning algorithm aimed to solve clustering problem with iterative calculation. This algorithm starts with a group of centroids, which are K instances randomly selected from the original dataset as the beginning points of every cluster. For every instance, we calculate its distance from every centroid and assign it to the nearest cluster (i.e. its distance from the centroid of this cluster is shorter than that from others). Once an instance is assigned to a certain cluster, we re-select the centroid from all the instanced in this cluster. This iteration goes on until either no (or minimal) instance remains to be assigned or no (or minimal) centroid moves. After the training of iteration, once fed with an instance, the algorithm assign it to the nearest cluster [75]. Despite the simplicity and speed, the K-means technique is limited in terms of robustness, since the clusters are determined by the initial random assignments [76].

#### Random forest (RF)

The random forest (RF) method proposed by Breiman [77] is a supervised learning algorithm based on decision trees, which is used in credit scoring, *inter alia*, imbalanced dataset [45]. The term “forest” refers to the model built on multiple decision trees. The term “random” indicates that the training set and the test features are selected randomly. Thus, this algorithm will not overfit [78], provided enough trees in the forest. We introduce the procedures as follows.

First, we randomly bootstrap m samples with replacement and acquire n training sets after n times of such bootstrapping (bagging). Second, we train the decision tree with each training set. Third, we split every decision tree using information gain or Gini importance to calculate the root node. Fourth, the forest chooses the classification with the most votes (each tree votes for a certain class) and the mean value of trees’ prediction is the prediction of the forest (each tree predicts class probabilities).

Combining multiple independent models, the random forest resists the problem of overfitting and noise. Furthermore, this approach incorporating a multitude of features is excellent when faced with high-dimensional data and is ready to detect underlying non-linear characteristics. Besides, the random forest is renowned for its speed of implementation.

### Swarm intelligence algorithm

As for the problem with no solution in traditional optimization based on individual agent and criterion, the swarm intelligence algorithm handle them by mimicking the natural biological evolution and/or the social behaviour of species. The systematic and organizational principles underlying individual and/or in-group behaviour of species are the core mechanism of these approaches. For example, herds and flocks cooperate in the search of food or mate. Every individual in the herd or flock learns from experience of other members as well as itself and adjusts its strategy of search accordingly.

#### Bat algorithm (BA)

Bat algorithm [79], aimed at global optimization, mimics the echolocation of bats. Assume that

1. All bats sense distance with echolocation and distinguish objectives from obstacles.
2. The bat flies randomly from point x_*i*_ at a speed of v_*i*_. Meanwhile, it makes a sound of fixed frequency f_min_, variant wavelength λ and volume. According to the distance from objective, the bat adjust the wavelength and transmission frequency *γ* ∈ [0, 1].
3. The volume *A*_0_ changes from the maximum to the fixed minimum.

The optimization in BA approach mimic the motion and food-seeking process of bats. Thus, The BA approach maps individual bats as feasible solutions in the space of high-dimension problems. The location of bat is assessed with the fitness function of objective and the solution is identified with recursions.

#### Cuckoo search optimization (CSO)

The cuckoo search optimization (CSO) algorithm [80] mimic the brood parasitism of cuckoo. Moreover, instead of random walk, the search process of CS algorithm mimics the discrete exploration of Lévy fly, composed by a series of straight motions and abrupt turning of 90 degree. Assume that

1. Every cuckoo lays one egg and randomly incubate it in a host nest.
2. The best nest with high-quality egg is passed onto the next generation.
3. The number of available host nest is fixed and the host detects cuckoo’s egg with a probability of p ∈ (0, 1). Once detecting the invading egg, the host either destroys it or discards the nest.

The CS approach is widely used in social science thanks to the efficient optimization with few parameters.

#### Firefly algorithm (FA)

The firefly algorithm [81] mimics the behavior of fireflies who flash for food and mate. With an explicit logic, the FA converge quickly to the global optimality.

The algorithm take the brightness of firefly as the objective value and assumes that

1. The attractiveness of a firefly is positively related with its brightness; i.e. the less bright one moves towards the brighter one.
2. The brightness is negatively related with the distance between two fireflies.
3. A firefly moves randomly if it detects no one brighter.

#### Gravitational search (GS) algorithm

The gravitational search algorithm [82] assumes search agents as masses obeying the Newtonian laws of gravitation and motion as follows.

Law of gravitation: agents attract each other, while the gravity between two agents is directly proportional to the product of their masses and inversely proportional to the distance between them.

Law of motion: the velocity of each agent keeps constant unless an external force acts upon it and the change of velocity equals to the force divided by the mass of the agent.

Thus, agents moves in accordance with the two laws above until the optimal position is reached.

#### Gray wolf optimization (GWO) algorithm

The gray wolf optimization algorithm mimics the hunting mechanism of gray wolves in nature [83]. Wolves keep strict social hierarchy represented by the division of a pack into four levels, each with different authority and responsibility.

Gray wolves hunt in three steps:

1. Tracking, chasing, and approaching the prey;
2. Pursuing, encircling, and harassing the prey until it stops moving;
3. Attack towards the prey.

#### Particle swarm optimization (PSO) algorithm

Particle swarm optimization (PSO) algorithm [84] is inspired by the predation of birds. Using massless particles moving in solution space, this algorithm mimics birds seeking food. Every particle finds the optimal solution on its own, known as local extremum, and shares the solution with all other particles. The global optimality is the best local extremum. Comparing local extremum and the global optimality, every particle adjust its motion in terms of direction and velocity. As a comparatively simple algorithm, the PSO approach is efficient in seeking optimality and is applicable to problems with real values.

#### Social spider algorithm (SSA)

Social spider algorithm [85] mimics the social spiders colony behavior. The SS algorithm assumes that

1. The search space is communal web of spiders where individuals could interact with each other.
2. Each solution within the search space represents a spider position in the communal web and each social spider is weighted according to the fitness value of the solution.
3. The spider colony is highly female-biased population with predefined proportion. Every social spider is assigned a set of cooperative behaviors according to its gender, known as evolutionary operators.

#### Whale swarm algorithm (WSA)

The whale swarm algorithm [86] mimics predation of humpback whales who seek food in a cooperative manner. This algorithm is recursive as follows.

1. All the whales communicate with each other by ultrasound in the search space.
2. Each whale has a certain degree of computing ability to calculate the distance to others.
3. The quality and quantity of food found are associated to the fitness of whale’s objective value.
4. A whale follows the nearest one with a better fitness.

## A novel framework: SI algorithm based BP-ANN credit-scoring model

**Fig 1:**
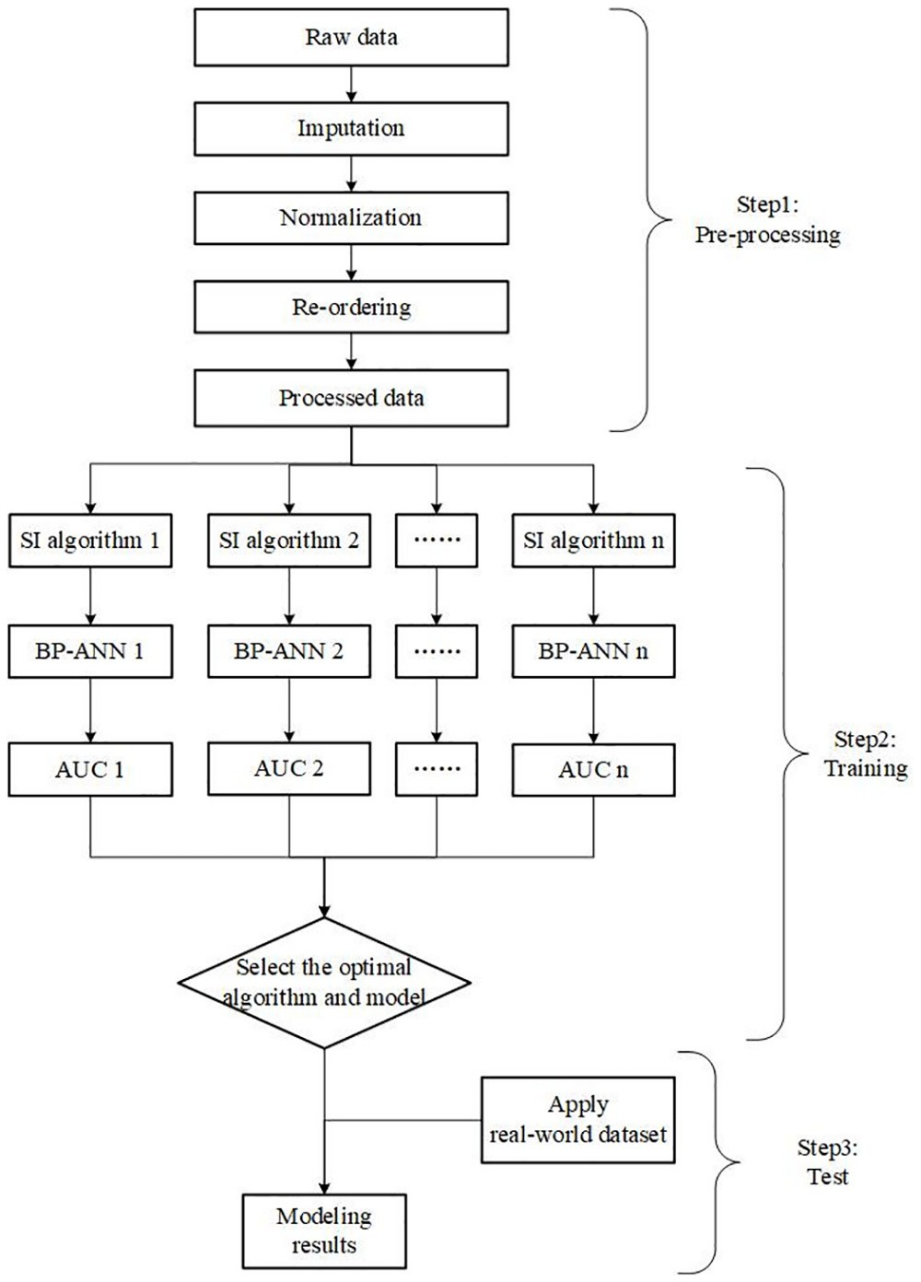
Flowchart of the framework. Plots the framework of this paper in three procedures: Pre-processing, optimization, and training.

In this section, we propose our novel framework based on swarm intelligence algorithm and BP-ANN model. The flowchart in Fig 1 plots the three procedures of constructing such framework: pre-processing, training, and test. In the first step, imputation, normalization and re-ordering are conducted to make the datasets suitable for further modelling. In the second step, we optimize the hyper-parameters of BP-ANN with swarm intelligence algorithm to find out the optimal model and the corresponding SI algorithm that suits specific scenarios and datasets. In the third step, we apply the BP-ANN model with the optimal parameters to evaluate the credit of new samples.

### Step 1: Pre-processing

#### Imputation

Missing values are prevalent in real-world credit-scoring datasets, partly owing to the insufficient information collection in business process or the careless of business staff and data manager. First, we identify the label (good or bad, default or not default) of each sample including missing values (samples with missing labels are dropped). Second, we select all the other samples with the same label whose values for the same attributes are not missing and assign them to the control group. Third, the missing values are replaced by the corresponding attribute of a sample randomly selected from the control group.

The above-mentioned imputation is comparatively efficient, for the time it consumes are proportional to the sample size of the dataset. Besides, for any specific attribute, the values with higher frequency (indicating strong connection with samples of the same label) are more likely to be selected in imputation, which helps to predict the label in the modelling stage.

#### Normalization

Although some models remain robust despite data scaling, distance-based models (e.g. KNN) are heavily dependent on attitude standardization. Considering the errors of each model are the same after normalization, the scaled dataset makes the comparison between different models possible. We employ the most commonly used scaling method shown in Eq (9).

$$x^{\prime }=\frac{x-\text{min}(X)}{\text{max}(X)-\text{min}(X)}$$ (9)

where *X* represents the vector of a specific attitude; *x* is the value of the attribute of one sample; and *x*′ is the scaled value of the same attribute in the sample.

#### Re-ordering

The order of samples matters. In some cases, the order of samples with different labels could affect the performance of sequential learning models. Furthermore, the imbalance sample problem might occur during the k-fold modelling process: if the good (or bad) samples make up a larger proportion in one dataset, the performance is biased across different datasets. It is easier for models to identify the pattern of samples labelled with dominant value, which causes unstable performance of models.

Before modelling, we re-order samples in accordance with the following procedures. First, samples are divided into two cohorts based on the binary label, denoted as “majority” and “minority” (dependent on the sample proportion). Second, we count the number of samples in each group with different labels and calculate the ratio of majority to minority. For example, the ratio for a dataset with 100 bad samples (minority) and 300 good samples (majority) is three. The ratios are round to integer. Third, we conduct sampling without replacement from two groups according to the proportion of each group in the population. Consider the context with three as the ratio. Three good samples are selected first, with one bad sample selected following; then another three good samples followed by one bad sample; etc. Finally, the samples left in the two groups after the above-mentioned sampling are assigned to the end sequence. Hence, all the samples are re-assigned to a predetermined sequence and the samples from two groups are distributed with more balance.

### Step 2: Training

We need to determine the optimal SI algorithm before using it to find out the optimal parameter set for BP-ANN model. However, the “grail algorithm” does not exist and we search for the optimal SI algorithm for heterogeneous dataset. First, we construct an alternative algorithm pool with several typical and widely used SI algorithms. For each SI algorithm in the pool, we construct a comparability scenario and set the same key hyper-parameters, including the number of individuals and time of iterations. Next, we set the feasible parameter space of BP-ANN model according to prior literature, including the size of hidden layers, the learning rate, the max iteration limit, and the tolerance of errors. Specifically, we set only the upper and lower boundary limit of each parameter and have SI algorithms to search the optimal parameters in the space. Finally, we apply SI algorithms in the pool one by one to optimize the parameter set of BP-ANN model (whose performance is sensitive to parameters) and find out the optimal SI algorithm with the highest value of area under curve (AUC) indicator (see the next section for further details). The BP-ANN model that optimized by the optimal SI algorithm is the core of our model.

### Step 3: Test

In this step, we apply the BP-ANN model whose parameters optimized by the optimal SI algorithm to another real-world dataset. We employ the data pre-processed in Step 1 and test the BP-ANN model with hyper-parameters determined in Step 2.

## Methodology

This paper carries out an experiment to test whether the BP-ANN model trained by swarm intelligence algorithm (treated group) outperforms prevalent classical models (control group) and several typical hybrid or ensemble models constructed in recent literature [29–34] within the context of credit scoring. First, the experiment investigates the performance of seven swarm intelligence algorithms (see Section “Swarm intelligence algorithm”) as an optimizer of BP-ANN model in the context of different datasets. Second, we compare the performance of trained BP-ANN models and those in control group, in order to classify the best in terms of generalization and robustness. Third, we analyze how the number of hidden layers affects the performance of trained BP-ANN models. Last, the time complexity of trained BP-ANN models is analyzed.

### Data

The instances of this paper are extracted from four public datasets of UCI (University of California, Irvine) and one dataset HELOC about credit scoring: first, the German Credit Dataset (the German dataset, https://archive.ics.uci.edu/ml/datasets/Statlog+%28German+Credit+Data%29); second, the Australian Credit Approval Dataset (the Australian dataset, https://archive.ics.uci.edu/ml/datasets/Statlog+%28Australian+Credit+Approval%29); third, the Japanese Credit Dataset (the Japanese dataset, https://archive.ics.uci.edu/ml/datasets/Credit+Approval); forth, the Default of Credit Card Clients Dataset from Taiwan (the Taiwan dataset, https://archive.ics.uci.edu/ml/datasets/default+of+credit+card+clients); and fifth, the Home Equity Line of Credit Dataset from the U.S. (the HELOC dataset, https://community.fico.com/s/explainable-machine-learning-challenge).

The reasons for these datasets selection are as follows. First, because of the unavailable dataset of commercial banks [87], public dataset is widely used in prior literature and results from the same dataset are comparable. Second, the German Credit Dataset (over 500 thousand page views), the Australian Credit Approval Dataset (over 155 thousand page views), the Japanese Dataset (over 393 thousand page views) and the Taiwan Dataset (over 350 thousand page views) are the most widely used datasets of UCI public credit scoring, with which a large body of prior literature study the performance of various models [22, 88]. Third, we focus on the diversity of datasets. On one hand, there are significant differences in the number of samples and attribute dimensions. On the other hand, these five datasets are extracted from five different financial markets (Germany, Australia, Japan, Taiwan and the US). Thus, we test not only the performance of each algorithm with different sample sizes and dimensions but also the feasibility of each algorithm in real world with different financial risks. The summary of datasets is presented in Table 1.

**Table 1:** Summary of datasets.

Dataset	Samples	Features	Good/Bad
German	1000	24	700/300
Australian	690	14	307/383
Japan	690	15	307/383
Taiwan	6000	23	3000/3000
HELOC	10459	23	5000/5459

Summarizes the variables in the datasets.

### Model evaluation

In line with the evaluation techniques proposed by recent literature [79], We select eight indicators (i.e. AUC, *accuracy*, *precision (pos)*, *precision (neg)*, *sensitivity*, *specificity*, Brier score, and G-mean) and the confusion matrix is presented in Table 2.

**Table 2:** Confusion matrix.

	Positive predictions	Negative predictions	Total
Actual positive instance	TP	FN	TP+FN
Actual negative instance	FP	TN	FP+TN
Total	TP+FP	FN+TN	TP+FN+FP+TN

Reports the confusion matrix where FP denotes the number of false positive predictions; TN denotes the number of actual negative instances; TP denotes the number of actual positive instances; and FN denotes the number of false negative predictions.

Based on the confusion matrix in Table 2, we construct five evaluation indicators as shown in Eqs (10) to (14).

$$accuracy=\frac{TP+TN}{TP+FN+FP+TN}$$ (10)

$$precision\left(pos\right)=\frac{TP}{TP+FN}$$ (11)

$$precision\left(neg\right)=\frac{TN}{FP+TN}$$ (12)

$$sensitivity=\frac{TP}{TP+FP}$$ (13)

$$specificity=\frac{FN}{FN+TN}$$ (14)

The indicator *accuracy* in Eq (10) measures the ratio of correctly identified sample over the entire sample. The indicator *precision (pos)* in Eq (11) measures the ratio of correctly identified positive sample over the entire positive sample, which evaluates how the model performs when classifying positive samples. Similarly, the indicator *precision (neg)* in Eq (12) measures the ratio of correctly identified negative sample over the entire negative sample, which evaluates how the model performs when classifying negative samples. The indicator *sensitivity* in Eq (13) measure the ratio of positive samples that are correctly predicted over the entire sample predicted as positive, which evaluates how precisely the model predicts in terms of positive samples. Similarly, the indicator *specificity* in Eq (14) measures the ratio of negative samples that are correctly predicted over the entire sample predicted as negative, which evaluates how precisely the model predicts in terms of negative samples.

To evaluate the comprehensive performance of each model, we employ the method of AUC (see details below). Using only one indicator, this method measures the classification ability of entire sample and the balance of classified samples simultaneously. The AUC builds on the knowledge of confusion matrix (see Table 2). We introduce the procedures of the AUC as follows. All predictive positive (default) probabilities of the model make the sequence *P*. The actual positive (default) probability works as threshold of classifier; i.e. the instance is classified as default if the predictive positive probability is larger than the threshold. Then we have the false positive rate and true positive rate as shown in Eqs (15) and (16).

$$false\;positive\;rate=\frac{FP}{FP+TN}$$ (15)

$$true\;positive\;rate=\frac{TP}{TP+FN}$$ (16)

**Fig 2:**
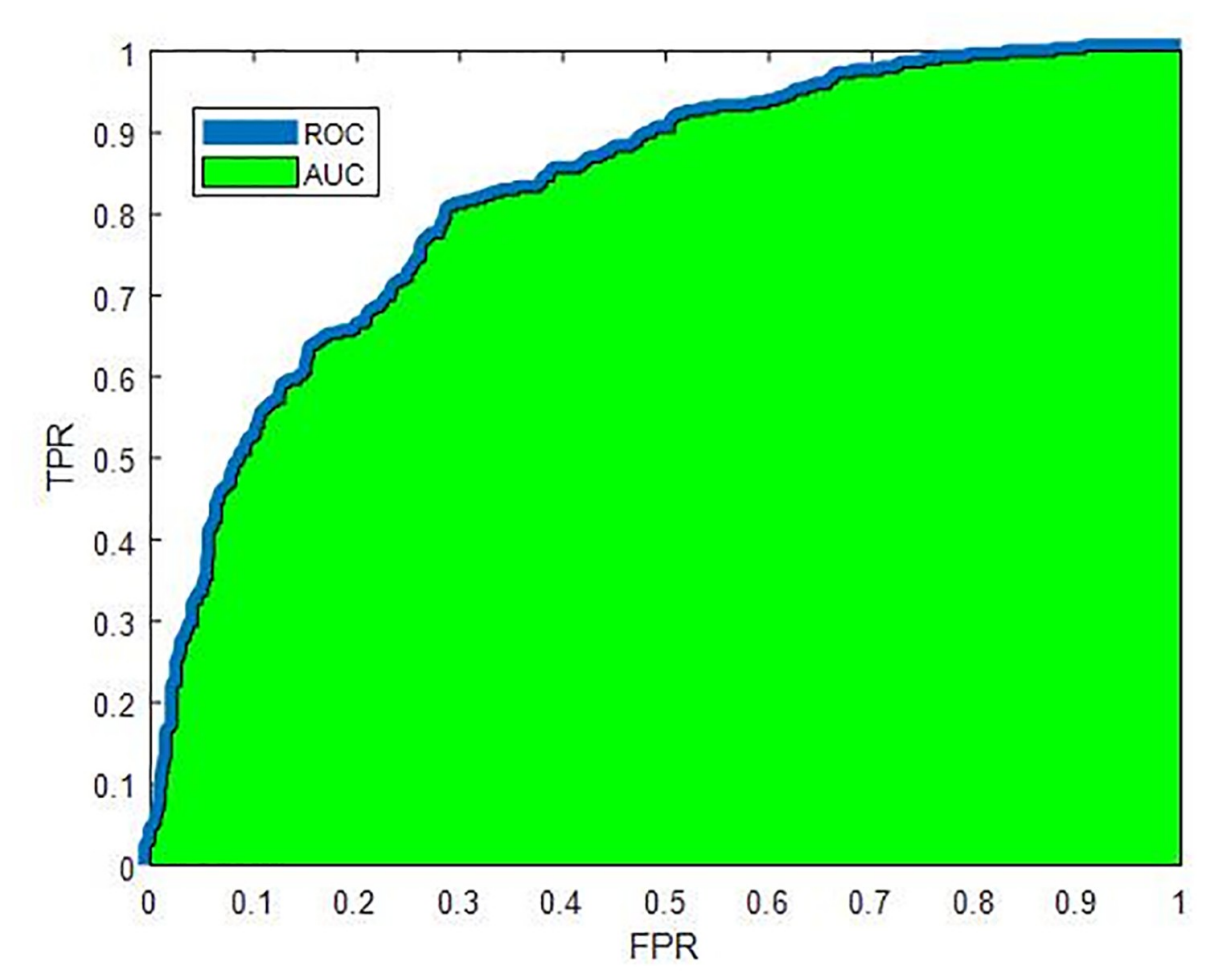
Area under the ROC curve (AUC). Plots the receiver operating characteristic (ROC) curve (see the blue curve), where the horizontal axis denotes the sequence of false positive rate and the vertical axis denote the sequence of true positive rate The area under the ROC curve (AUC) indicates the performance of model as a classifier (see the green part).

Thus, we have two sequences: sequence of false positive rates and sequence of true positive rates. After that, with false positive rate sequence as the x-axis while true positive rate sequence as the y-axis, we draw the curve of receiver operating characteristic (ROC) (see Fig 2) and the area under the ROC curve (area under curve, AUC) is positively related with the performance of classification model.

In real word, the datasets are usually imbalanced (e.g., good samples make up a greater proportion than bad samples) and three principled evaluation metrics (i.e. the Brier score, G-mean, and H-measure) are introduced thereby. The H-measure requires predetermined distribution of misclassification cost [89] and is less prevalent in recent evaluation. Thus, we use the other two metrics (i.e. the Brier score, and G-mean) simultaneously to evaluate the performance of each model with the imbalanced datasets.

As shown in Eq (17), the Brier score measures the mean square error of predicted and true value.

$$Brier\;score=\frac{1}{N}*\sum_{i=1}^{N}{\left(p_{i}-t_{i}\right)}^{2}$$ (17)

where *p*_*i*_ and *t*_*i*_ are the predicted value and true value, respectively; and *N* is the sample size.

With the result of Eqs (13) and (14), the G-mean is measured as show in Eq (18).

$$G-mean=\sqrt{sensitivity*specificity}$$ (18)

### Settings

First, this paper trains parameters of the BP-ANN model with the seven approaches of swarm algorithm described in Section “Swarm intelligence algorithm”. Considering the time complexity and accuracy, each swarm algorithm contains 10 individuals and iterates 20 times. The neural network contains one input layer, one hidden layer, and one output layer from the beginning. In the later stage, we increase the number of hidden layers in BP-ANN. Parameters of the neural network are trained in the parameter space as presented in Table 3.

**Table 3:** Parameters of neural network.

Optimization parameter	Min	Max	Type
Number of neurons in the hidden layer	10	200	int
Learning rate	0.0001	0.1	float
Maximum number of iterations in the network	20	500	int
Maximum fault tolerance	0.001	0.1	float

Reports the parameters of neural network trained by swarm algorithm approaches.

The procedures that we optimize the BP-ANN model with swarm intelligence algorithm are introduced as follows.

Step 1: 10 four-dimension feasible solutions are randomly selected from the solution space (The four dimensions are the number of neurons in the hidden layer, the learning rate, the maximum number of iterations in the network, and the maximum fault tolerance. The value range of each dimension is shown in Table 5). Training the BP-ANN model with the parameter set of each feasible solution, we have 10 models with different parameters. After evaluation, we take the best performance as the “present global optimal value” of the certain swarm intelligence algorithm and the certain feasible solution as the “present global optimal solution”.
Step 2: based on the optimization mechanism and principles of the certain swarm intelligence algorithm, we set out from the present feasible solution and start an exploration of “novel feasible solution”. If the “novel feasible solution” is better than the present feasible solution, we replace the present feasible solution with the “novel feasible solution” and compare the novel one with the “present global optimal solution”. If the novel one is better, we replace the “present global optimal solution” with the “novel feasible solution” and take the performance of the novel one as the “present global optimal value”.
Step 3: if the stopping condition of the certain swarm intelligence algorithm is not achieved, then we repeat Step 2. Otherwise, we stop the optimization and take the “present global optimal solution” as the final solution. Then we set parameters of BP-ANN accordingly.

In line with the prior literature, this paper applies 5-fold cross validation. To be specific, all the instances from datasets are divided into five pairs of training-test sets. For each pair, training set predicts parameters and constructs model accordingly. Then we examine the generalization of model using the test set in order to decide whether it fits new instances that isolated from the train set. The process runs five times to ensure the model is robust. All models introduced in Section “Prevalent classical models for credit scoring” (i.e. logistic regression, NB approach, DA, KNN, DT, SVM, K means, and RF) are enrolled in control group. Within the context of the same public datasets, we enroll several typical hybrid or ensemble models proposed in recent literature [29–34] in the control group. When evaluating the performance of difference models, we report the value of eight indicators in Section “Model evaluation” while focusing on the value of AUC (see Section “Model evaluation” for detailed calculation). As is mentioned above, the AUC not only reflects the entire precision of the model but also indicates how the model performs when classifying a certain category of instances.

Models in Section “Prevalent classical models for credit scoring” are constructed with the build-in package of Matlab 2017a to build up while the “Optimize Hyperparameters” is set as “all”. As the command indicates, a body of parameter sets including the kernel functions of SVM model are applied to each baseline model. Notably, we carry out all the experiments in this study using a PC of 3.4 GHz, Intel CORE i5-7500 and 8GB RAM with the operating system of Microsoft Windows 10.

## Findings

First, we employ seven swarm intelligence algorithms to train BP-ANN and report the performance of trained models in the first subsection “Optimization”. Further, the performance of control group is reported and compared in the second subsection “Control group”. We also present the performance of our model while hidden layers of the BP-ANN increasing, followed by analysis of computational complexity (i.e. runtime). Last, by comparison with control group, we show how our framework balances between accuracy and efficiency.

### Optimization

Table 4 reports the performance of BP-ANN models with the five datasets described in Section “Data”, where parameters are trained by different swarm algorithms. The value of eight indicators in Section “Model evaluation” are reported while the optimal performance are presented in bold.

**Table 4:** Performance of BP-ANN trained by different swarm intelligence algorithms.

Dataset	Phase	Indicator	*BA*	*CSO*	*FA*	*GS*	*GWO*	*PSO*	*WSA*
German	Training	*AUC*	0.8037	**0.8859**	0.8135	0.8052	0.8150	0.8641	0.8656
		*accuracy*	0.7605	**0.8253**	0.7795	0.7770	0.7720	0.8143	0.8108
		*precision (pos)*	0.3659	0.5671	0.4409	0.5123	0.4290	0.5413	**0.5674**
		*precision (neg)*	0.9282	**0.9361**	0.9242	0.8910	0.9197	0.9311	0.9149
		*sensitivity*	0.6937	**0.7934**	0.7190	0.6670	0.6984	0.7713	0.7443
		*specificity*	0.7770	**0.8348**	0.7947	0.8110	0.7905	0.8259	0.8319
		Brier score	0.2395	**0.1748**	0.2205	0.2230	0.2280	0.1858	0.1893
		G-mean	0.5639	**0.7279**	0.6367	0.6713	0.6238	0.7095	0.7196
	Testing	*AUC*	0.7864	0.7860	0.7712	0.7681	0.7843	**0.8004**	0.7916
		*accuracy*	0.7460	0.7620	0.7460	0.7450	0.7550	**0.7660**	0.7630
		*precision (pos)*	0.3458	0.4321	0.3942	0.4636	0.3834	0.4533	**0.4804**
		*precision (neg)*	**0.9233**	0.9025	0.8978	0.8629	0.9124	0.9006	0.8845
		*sensitivity*	**0.6918**	0.6602	0.6263	0.6007	0.6596	0.6686	0.6355
		*specificity*	0.7678	0.7880	0.7757	0.7895	0.7762	0.7947	**0.8006**
		Brier score	0.2540	0.2380	0.2540	0.2550	0.2450	**0.2340**	0.2370
		G-mean	0.5514	0.6227	0.5939	0.6305	0.5886	0.6357	**0.6443**
Australian	Training	*AUC*	0.9395	**0.9514**	0.9324	0.9421	0.9432	0.9460	0.9432
		*accuracy*	0.8659	**0.8783**	0.8612	0.8685	0.8728	0.8761	0.8728
		*precision (pos)*	0.9169	0.9047	0.9138	0.9110	0.9084	0.9097	**0.9186**
		*precision (neg)*	0.8250	**0.8570**	0.8193	0.8342	0.8436	0.8493	0.8362
		*sensitivity*	0.8080	**0.8355**	0.8024	0.8150	0.8249	0.8290	0.8181
		*specificity*	0.9256	0.9183	0.9224	0.9217	0.9210	0.9216	**0.9277**
		Brier score	0.1341	**0.1217**	0.1388	0.1315	0.1272	0.1239	0.1272
		G-mean	0.8697	**0.8805**	0.8651	0.8717	0.8750	0.8788	0.8763
	Testing	*AUC*	0.9289	0.9301	0.9229	0.9351	**0.9373**	0.9326	0.9305
		*accuracy*	0.8623	0.8565	0.8565	**0.8638**	0.8609	0.8609	0.8580
		*precision (pos)*	**0.9164**	0.8744	0.8982	0.9020	0.9026	0.8918	0.8998
		*precision (neg)*	0.8203	**0.8438**	0.8182	0.8388	0.8288	0.8346	0.8256
		*sensitivity*	0.8050	**0.8175**	0.7997	0.8125	0.8052	0.8173	0.8030
		*specificity*	**0.9237**	0.8948	0.9164	0.9144	0.9121	0.9090	0.9100
		Brier score	0.1377	0.1435	0.1435	**0.1362**	0.1391	0.1391	0.1420
		G-mean	0.8664	0.8579	0.8563	**0.8691**	0.8648	0.8610	0.8617
Japanese	Training	*AUC*	0.9417	0.9354	0.9427	0.9402	0.9408	**0.9455**	0.9425
		*accuracy*	**0.8736**	0.8699	0.8728	0.8692	0.8699	0.8725	0.8728
		*precision (pos)*	0.8322	0.8210	0.8294	**0.8322**	0.8204	0.8292	0.8289
		*precision (neg)*	0.9251	0.9307	0.9264	0.9154	**0.9316**	0.9266	0.9275
		*sensitivity*	0.9328	0.9368	0.9346	0.9258	**0.9374**	0.9341	0.9347
		*specificity*	**0.8156**	0.8070	0.8136	0.8149	0.8064	0.8125	0.8135
		Brier score	**0.1264**	0.1301	0.1272	0.1308	0.1301	0.1275	0.1272
		G-mean	**0.8773**	0.8740	0.8764	0.8724	0.8741	0.8765	0.8766
	Testing	*AUC*	0.9202	0.9254	0.9238	0.9253	**0.9354**	0.9214	0.9238
		*accuracy*	0.8507	**0.8623**	0.8522	0.8580	**0.8623**	0.8536	0.8536
		*precision (pos)*	0.8051	0.8070	0.8035	**0.8149**	0.8116	0.8102	0.8107
		*precision (neg)*	0.9051	**0.9280**	0.9111	0.9118	0.9267	0.9116	0.9059
		*sensitivity*	0.9138	**0.9334**	0.9191	0.9202	0.9302	0.9191	0.9134
		*specificity*	0.7898	**0.7978**	0.7891	0.7970	0.7978	0.7885	0.7961
		Brier score	0.1493	**0.1377**	0.1478	0.1420	**0.1377**	0.1464	0.1464
		G-mean	0.8533	0.8648	0.8553	0.8618	**0.8672**	0.8589	0.8567
Taiwan	Training	*AUC*	**0.7596**	0.7466	0.7139	0.7356	0.7594	0.7389	0.7410
		*accuracy*	**0.6962**	0.6849	0.6461	0.6697	0.6934	0.6794	0.6756
		*precision (pos)*	0.5772	0.5702	**0.6459**	0.6224	0.5804	0.5767	0.5826
		*precision (neg)*	**0.8152**	0.7997	0.6466	0.7171	0.8060	0.7823	0.7689
		*sensitivity*	**0.7585**	0.7448	0.6555	0.6929	0.7535	0.7313	0.7324
		*specificity*	**0.6587**	0.6509	0.6477	0.6560	0.6583	0.6499	0.6530
		Brier score	**0.3038**	0.3151	0.3539	0.3303	0.3066	0.3206	0.3244
		G-mean	**0.6856**	0.6735	0.6398	0.6651	0.6824	0.6692	0.6600
	Testing	*AUC*	**0.7403**	0.7271	0.7070	0.7233	0.7396	0.7230	0.7242
		*accuracy*	**0.6852**	0.6737	0.6397	0.6617	0.6810	0.6730	0.6647
		*precision (pos)*	0.5625	0.5578	**0.6404**	0.6145	0.5673	0.5647	0.5716
		*precision (neg)*	**0.8075**	0.7893	0.6376	0.7085	0.7968	0.7808	0.7570
		*sensitivity*	**0.7457**	0.7318	0.6488	0.6827	0.7392	0.7253	0.7156
		*specificity*	0.6492	0.6429	0.6419	0.6483	**0.6494**	0.6425	0.6404
		Brier score	**0.3148**	0.3263	0.3603	0.3383	0.3190	0.3270	0.3353
		G-mean	**0.6732**	0.6599	0.6310	0.6572	0.6699	0.6621	0.6514
HELOC	Training	*AUC*	0.7902	0.7926	0.7826	0.7928	0.7915	**0.7936**	0.7890
		*accuracy*	0.7167	0.7157	0.7081	0.7172	0.7191	**0.7212**	0.7167
		*precision (pos)*	0.7278	0.7512	0.7410	0.7466	0.7650	0.7624	**0.7826**
		*precision (neg)*	**0.7047**	0.6768	0.6727	0.6849	0.6693	0.6757	0.6449
		*sensitivity*	**0.7297**	0.7210	0.7164	0.7235	0.7174	0.7209	0.7086
		*specificity*	0.7045	0.7193	0.7125	0.7161	0.7254	0.7247	**0.7350**
		Brier score	0.2833	0.2843	0.2919	0.2828	0.2809	**0.2788**	0.2833
		G-mean	0.7154	0.7093	0.7007	0.7128	0.7142	**0.7166**	0.7080
	Testing	*AUC*	0.7858	0.7880	0.7809	0.7866	0.7867	**0.7898**	0.7867
		*accuracy*	0.7132	0.7128	0.7095	0.7115	0.7186	**0.7205**	0.7126
		*precision (pos)*	0.7206	0.7465	0.7422	0.7421	0.7672	0.7593	**0.7796**
		*precision (neg)*	**0.7047**	0.6761	0.6719	0.6789	0.6643	0.6793	0.6388
		*sensitivity*	**0.7274**	0.7182	0.7158	0.7192	0.7155	0.7214	0.7048
		*specificity*	0.6992	0.7172	0.7121	0.7109	0.7254	0.7217	**0.7307**
		Brier score	0.2868	0.2872	0.2905	0.2885	0.2814	**0.2795**	0.2874
		G-mean	0.7120	0.7067	0.7017	0.7066	0.7125	**0.7176**	0.7028

Reports the performance of BP-ANN models with the five datasets (i.e. German Credit Dataset, Australian Credit Approval Dataset, Japanese Credit Dataset, Taiwan Credit Dataset and HELOC Dataset). Parameters are trained by seven different swarm algorithms (i.e. BA, CSO, FA, GS, GWO, PSO, and WSA). Eight indicators (i.e. the AUC, *accuracy*, *precision (pos)*, *precision (neg)*, *sensitivity*, *specificity*, Brier score, and G-mean) are reported as evaluation metrics. The bold text indicates the best performance of the row.

First, we compare the model performance with the German Credit Dataset, as shown in the first 12 rows of Table 6. In the training phase, the fitness of CHSO-BP-ANN model scores highest in terms of all six indicators, which implies that the fitting ability of this model is best and robust. The fitness of PSO-BP-ANN, WSA-BP-ANN, and BA-BP-ANN performs less competent but still acceptable, with the AUC value over 0.9. Focused on the classification of positive samples, we notice that all the models except CHSO-BP-ANN and PSO-BP-ANN score no more than 0.8 in terms of *precision (pos)*. However, all the models performs better when classifying negative, with the value of *precision (neg)* over 0.9. The classification is biased towards the negative samples owing to the fact that, in the training set, the size of negative sample set is far larger than that of positive sample set. In the phase of test, the model CSO-BP-ANN performs best in terms of AUC, *accuracy* and *precision (neg)*. The model CHSO-BP-ANN, performing well in the training phase though, scores highest in terms of *precision (pos)* and *specificity* in the testing phase, which indicates excellent classification of positive sample and precise prediction. Besides, the model SSA-BP-ANN and FA-BP-ANN classify comparatively precisely.

As shown in Table 4, we first compare the model performance with the German dataset. In the training phase, the CSO-BP-ANN model in training phase scores the highest in terms of all indicators except the *precision (pos)*. It indicates that the CSO-BP-ANN performs best when distinguishing samples with opposite attributes from each other. In addition, the WSA-BP-ANN scores the highest in terms of the *precision (pos)* (0.5674), indicating that this model performs best when identifying the positive samples. The performance across models differs within a comparatively limited range in terms of the AUC (approx. 0.08) and *accuracy* (approx. 0.06). Besides, the overall performance of PSO-BP-ANN and GS-BP-ANN is strong in terms of all indicators while the performance of BA-BP-ANN and GS-BP-ANN is comparatively weak. In the testing phase, the PSO-BP-ANN performs best generalization in terms of overall classification and prediction with imbalanced datasets. To be specific, the PSO-BP-ANN scores 0.8004 for AUC, 0.7660 for the *accuracy*, and 0.2340 for the Brier score. Furthermore, the WSA-BP-ANN scores the highest in terms of *precision (pos)*, *specificity*, and G-mean, while BA-BP-ANN who performs moderately during training phase scores the highest in terms of *precision (neg)* and *sensitivity*.

Within the context of the Australian dataset, in the training phase, the CSO-BP-ANN scores the highest in terms of all indicators except *precision (pos)* and *specificity*. Similarly to the context of German dataset, the WSA-BP-ANN scores the highest in terms of *precision (pos)* and *specificity*. The performance of other models varies within a limited range in terms of the AUC (approx.0.02) and *accuracy* (approx. 0.02), indicating that BP-ANN trained by these SI algorithms presents a strong performance when identifying the classifiable attributes in the training set. In the testing phase, the GWO-BP-ANN and the BS-BP-ANN perform best in terms of the AUC (0.9373) and *accuracy* (0.8638), respectively. Meanwhile, the GS-BP-ANN performs best in terms of the Brier score and G-mean, indicating strongest balance of generalization among the models. Besides, the BA-BP-ANN and the CSO-BP-ANN present strong performance in terms of identifying the positive and negative samples.

Within the context of the Japanese dataset, in the training phase, the CSO-BP-ANN who performs best in the previous context scores the highest in terms of no indicator. The PSO-BP-ANN scores the highest in terms of the AUC (0.9455) while the BA-BP-ANN performs best in terms of *accuracy* (0.8736) as well as *specificity*, Brier score and G-mean. In addition, the GS-BP-ANN and the GWO-BP-ANN present strong performance when identifying the positive and negative samples. In the testing phase, the GWO-BP-ANN presents strong performance of overall and balance of prediction by scoring the best in terms of the AUC, *accuracy*, Brier score, and G-mean. Besides, the GWO-BP-ANN, despite the moderate performance in the training phase, scores the best in terms of *accuracy*, *precision (neg)*, *sensitivity*, *specificity*, and Brier score.

Within the context of the Taiwan dataset, in the training phase, the BA-BP-ANN performs best in terms of all indicators except *precision (pos)*, indicating excellent fitness of the training set. In terms of overall identification (i.e. the AUC and *accuracy*), the difference of performance between the BA-BP-ANN and the other models remains around 0.04 (for the AUC) and 0.05 (for *accuracy*). In terms of balance of identification (i.e. Brier score and G-mean), the difference remains around 0.05. In other word, the performance remains comparatively stable across different models. In the testing phase, the BA-BP-ANN performs still best in terms of all indicators except *precision (pos)* and *specificity*. The difference of performance between the BA-BP-ANN and the other models remains around 0.04 (for the AUC) and 0.05 (for *accuracy*) in terms of overall identification, and 0.05 (for Brier score) and 0.04 (for G-mean) in terms of balanced identification. Thus, all the models perform robustly through the two phases.

Finally, we compare the performance within the context of the HELOC dataset. In the training phase, the PSO-BP-ANN performs best in terms of overall and balanced fitting, with the best score in the AUC, *accuracy*, Brier score, and G-mean. Meanwhile, the BA-BP-ANN and the WSA-BP-ANN perform better when identifying samples with certain attributes. The performance of models varies within an average range of 0.0274. In the testing phase, the PSO-BP-ANN, the VA-BP-ANN, and the WSA-BP-ANN remain their highest scores in the training phase, which indicates robust performance of these models. The difference of performance across models remains in an average range of 0.0282, moderately greater than the range during training phase but still limited.

A further comparison of performance within the context of five datasets is conducted as follows.

#### First, volatility across models

With the five datasets, the average range of scores measures 0.1008 (German), 0.0203 (Australian), 0.0091 (Japanese), 0.0687 (Taiwan), and 0.0274 (FICO) during the training phase while 0.0608 (German), 0.0195 (Australian), 0.0145 (Japanese), 0.0656 (Taiwan), and 0.0282 (FICO) during the testing phase. Generally, the range of scores is limited, which indicates robustness across BP-ANN models trained by different SI algorithms.

#### Second, stability within model

The performance of models during the testing phase is slightly weaker than that during the training phase. To be specific, comparing the scores during the two stages, the difference measures no more than 0.01 for most of the indicators. In other word, the BP-ANN models trained by SI algorithms are robust across the training and testing phases and thereby are useful in the real world where practitioners select model based on the performance of training.

#### Third, the optimal SI algorithm

Unfortunately, we see no evidence that a model performs best with all the five datasets. For instead, the characteristics of each dataset affect how the SI algorithm optimizes the model. The BP-ANN model trained by the CSO, GS, GWO, BA, PSO, and WSA presents best performance in terms of different indicators within different context. That is why we propose the selection of SI algorithm and the framework of modelling in the section of “Methodology”. In the real world, due to the lack of knowledge when facing a new context, we have to search for the optimal SI algorithm rather than determine with prior knowledge. Furthermore, the search is feasible because of the stability within model.

### Control group

In this section, we compare the performance of the BP-ANN trained by SI algorithms with models in the control group. The control group includes classical credit scoring models mentioned in section “Prevalent classical models for credit scoring” (i.e. logistic regression, NB approach, DA, KNN, DT, linear and polynomial SVM, SVM-RBF, K means, and RF) and several hybrid or ensemble models constructed in recent literature [29–34].

Table 5 reports how the classical models (i.e. logistic regression, NB approach, DA, KNN, DT, linear and polynomial SVM, SVM-RBF, K means, and RF) perform within the context of five datasets (see the section “Data” for details). The value of evaluation metrics (see the section “Model evaluation” for details) are reported with the optimal performance (i.e. the lowest value for Brier score and the highest value for other indicators) presented in bold. Notably, as a lazy learning model, the KNN excludes the training phase.

**Table 5:** Performance of classical models in control group.

Dataset	Phase	Indicator	*DT*	*DA*	*Logistic*	*SVM*	*KNN*	*NB*	*k-means*	*RF*
German	Training	*AUC*	0.7438	0.8192	0.8208	0.8632	-	0.8111	0.5603	**0.9985**
		*accuracy*	0.7668	0.7835	0.7870	0.8303	-	0.7768	0.5920	**0.9993**
		*precision (pos)*	0.6765	0.6785	0.6927	0.7757	-	0.6579	0.3433	**1.0000**
		*precision (neg)*	0.7901	0.8154	0.8144	0.8448	-	0.8143	0.7221	**0.9989**
		*sensitivity*	0.4301	0.5283	0.5209	0.5964	-	0.5296	0.3860	**0.9975**
		*specificity*	0.9097	0.8922	0.9004	0.9297	-	0.8826	0.6812	**1.0000**
		Brier score	0.1661	0.1524	0.1517	0.1698	-	0.1569	0.2385	**0.0008**
		G-mean	0.7305	0.7438	0.7510	0.8089	-	0.7317	0.4959	**0.9995**
	Testing	*AUC*	0.7177	**0.8002**	0.7972	0.7762	0.7624	0.7817	0.5476	0.6605
		*accuracy*	0.7190	0.7640	**0.7660**	0.7550	0.7420	0.7500	0.6110	0.7630
		*precision (pos)*	0.5465	0.6385	0.6487	0.6262	**0.6622**	0.6145	0.3467	0.6524
		*precision (neg)*	0.7568	**0.8017**	0.8012	0.7894	0.7591	0.7940	0.7184	0.7888
		*sensitivity*	0.3359	**0.5084**	0.5053	0.4693	0.3258	0.4932	0.3258	0.4431
		*specificity*	0.8871	0.8804	0.8864	0.8855	**0.9269**	0.8678	0.7417	0.9028
		Brier score	0.1869	**0.1637**	0.1639	0.2450	0.1763	0.1724	0.2386	0.2370
		G-mean	0.6394	0.7123	**0.7177**	0.7012	0.7026	0.6939	0.4929	0.7145
Australian	Training	*AUC*	0.8879	0.9289	0.9421	0.9551	-	0.9043	0.5690	**0.9997**
		*accuracy*	0.8808	0.8746	0.8786	0.8906	-	0.8072	0.5627	**0.9996**
		*precision (pos)*	0.8600	0.8412	0.8474	0.8555	-	0.8624	**1.0000**	0.9992
		*precision (neg)*	0.9063	0.9038	0.9054	0.9262	-	0.7775	0.5593	**1.0000**
		*sensitivity*	0.8839	0.8846	0.8867	0.9127	-	0.6724	0.0171	**1.0000**
		*specificity*	0.8805	0.8656	0.8716	0.8716	-	0.9143	**1.0000**	0.9994
		Brier score	0.0941	0.1032	0.0904	0.1094	-	0.1615	0.4348	**0.0004**
		G-mean	0.8815	0.8719	0.8758	0.8897	-	0.8187	0.7476	**0.9996**
	Testing	*AUC*	0.8617	0.9267	**0.9279**	0.9222	0.9092	0.8944	0.6687	0.8452
		*accuracy*	0.8478	**0.8768**	0.8623	0.8522	0.8435	0.7986	0.6116	0.8754
		*precision (pos)*	0.7901	0.8345	0.8155	0.8005	**0.8373**	0.8310	0.6819	0.8282
		*precision (neg)*	0.8832	**0.8977**	0.8866	0.8865	0.8391	0.7663	0.6099	0.8970
		*sensitivity*	0.8427	**0.8850**	0.8698	0.8799	0.7967	0.6546	0.3286	0.8679
		*specificity*	0.8318	0.8724	0.8560	0.8349	0.8879	**0.9010**	0.8171	0.8714
		Brier score	0.1236	0.1052	**0.1028**	0.1478	0.1215	0.1668	0.3012	0.1246
		G-mean	0.8328	**0.8630**	0.8477	0.8391	0.8335	0.7950	0.6276	0.8600
Japanese	Training	*AUC*	0.8994	0.9228	0.9348	0.9276	-	0.8601	0.5378	**0.9987**
		*accuracy*	0.8717	0.8659	0.8775	0.8652	-	0.7870	0.5623	**0.9982**
		*precision (pos)*	0.8906	0.9347	0.9269	0.9396	-	0.7636	0.5591	**0.9987**
		*precision (neg)*	0.8567	0.8016	0.8272	0.7971	-	0.8366	**1.0000**	0.9976
		*sensitivity*	0.8799	0.8159	0.8464	0.8095	-	0.9001	**1.0000**	0.9980
		*specificity*	0.8594	0.9280	0.9157	0.9346	-	0.6455	0.0163	**0.9984**
		Brier score	0.0999	0.1066	0.0921	0.1348	-	0.1680	0.4328	**0.0018**
		G-mean	0.8729	0.8656	0.8756	0.8654	-	0.7990	0.7477	**0.9981**
	Testing	*AUC*	0.8916	**0.9189**	0.9177	0.9078	0.8991	0.8370	0.6409	0.8605
		*accuracy*	0.8391	0.8580	0.8551	0.8507	0.8174	0.7783	0.5826	**0.8594**
		*precision (pos)*	0.8597	0.9235	0.9028	**0.9294**	0.8439	0.7633	0.4766	0.8840
		*precision (neg)*	0.8224	0.7954	0.8048	0.7780	0.7891	0.8045	0.4446	**0.8287**
		*sensitivity*	0.8617	0.8139	0.8292	0.7932	0.8302	**0.8785**	0.7797	0.8622
		*specificity*	0.8192	0.9137	0.8893	**0.9237**	0.8052	0.6527	0.2718	0.8608
		Brier score	0.1182	0.1180	**0.1043**	0.1493	0.1295	0.1720	0.3775	0.1406
		G-mean	0.8379	**0.8560**	0.8513	0.8495	0.8142	0.7831	0.3505	0.8547
Taiwan	Training	*AUC*	0.6985	0.7032	0.7063	0.7395	-	0.7082	0.5115	**0.9956**
		*accuracy*	0.6812	0.6575	0.6548	0.6892	-	0.5805	0.5093	**0.9961**
		*precision (pos)*	0.7532	0.6771	0.6682	0.7683	-	0.5526	0.5053	**0.9926**
		*precision (neg)*	0.6419	0.6421	0.6435	0.6466	-	0.6724	0.5382	**0.9996**
		*sensitivity*	0.5405	0.6032	0.6153	0.5412	-	0.8461	0.8887	**0.9996**
		*specificity*	0.8219	0.7119	0.6943	0.8372	-	0.3148	0.1298	**0.9926**
		Brier score	0.2115	0.2166	0.2157	0.3108	-	0.2936	0.4061	**0.0039**
		G-mean	0.6952	0.6593	0.6557	0.7048	-	0.6095	0.5215	**0.9961**
	Testing	*AUC*	0.6910	0.6982	0.7010	0.7168	**0.7213**	0.7063	0.5475	0.6770
		*accuracy*	0.6727	0.6523	0.6498	0.6778	0.6717	0.5798	0.5233	**0.6783**
		*precision (pos)*	0.7415	0.6722	0.6637	**0.7553**	0.7105	0.5523	0.5178	0.6982
		*precision (neg)*	0.6354	0.6367	0.6382	0.6372	0.6469	**0.6696**	0.5391	0.6621
		*sensitivity*	0.5323	0.5950	0.6077	0.5273	0.5853	**0.8453**	0.7887	0.6283
		*specificity*	0.8130	0.7097	0.6920	**0.8283**	0.7580	0.3143	0.2580	0.7283
		Brier score	**0.2144**	0.2181	0.2175	0.3222	0.2166	0.2954	0.3167	0.3217
		G-mean	0.6863	0.6542	0.6508	**0.6936**	0.6778	0.6081	0.5284	0.6799
HELOC	Training	*AUC*	0.7388	0.7828	0.7841	0.7950	-	0.7973	0.6194	**0.9727**
		*accuracy*	0.7148	0.7175	0.7180	0.7287	-	0.7274	0.5881	**0.9745**
		*precision (pos)*	0.7136	0.7187	0.7177	0.7211	-	0.7387	0.5913	**0.9535**
		*precision (neg)*	0.7191	0.7159	0.7184	0.7388	-	0.7152	0.5836	**0.9997**
		*sensitivity*	0.7594	0.7536	0.7577	0.7831	-	0.7393	0.6836	**0.9998**
		*specificity*	0.6661	0.6780	0.6747	0.6692	-	0.7144	0.4840	**0.9468**
		Brier score	0.1988	0.1895	0.1891	0.2713	-	0.2181	0.2794	**0.0255**
		G-mean	0.7161	0.7173	0.7181	0.7299	-	0.7268	0.5874	**0.9764**
	Testing	*AUC*	0.7307	0.7808	0.7815	**0.7851**	0.7804	0.7786	0.6161	0.7092
		*accuracy*	0.7025	0.7165	0.7167	**0.7214**	0.7114	0.7145	0.5833	0.7132
		*precision (pos)*	0.7031	0.7188	0.7168	0.7157	0.7204	**0.7262**	0.6113	0.7081
		*precision (neg)*	0.7054	0.7140	0.7169	**0.7293**	0.7019	0.7018	0.5675	0.7198
		*sensitivity*	0.7487	0.7510	0.7565	**0.7743**	0.7319	0.7276	0.5689	0.7669
		*specificity*	0.6524	0.6790	0.6734	0.6637	0.6893	**0.7003**	0.6005	0.6545
		Brier score	0.2034	0.1905	**0.1902**	0.2786	0.1921	0.2289	0.2413	0.2868
		G-mean	0.7040	0.7164	0.7168	**0.7224**	0.7110	0.7139	0.5884	0.7139

Reports the performance of classical models (i.e. logistic regression, NB approach, DA, KNN, DT, linear and polynomial SVM, SVM-RBF, K means, and RF) with the five datasets (i.e. German Credit Dataset, Australian Credit Approval Dataset, Japanese Credit Dataset, Taiwan Credit Dataset and HELOC Dataset). Eight indicators (i.e. the AUC, *accuracy*, *precision (pos)*, *precision (neg)*, *sensitivity*, *specificity*, Brier score, and G-mean) are reported as evaluation metrics. The bold text indicates the best performance of the row. The KNN is a lazy learning model and the training phase is excluded thereby.

As is shown in Table 5, we first focus on the performance of classical models within the context of the German dataset. During the training phase, the RF model outperforms all the other competing models in terms of all indicators. As for most indicators, the evaluation is “nearly perfect” (i.e. with a value very close to 1 or to 0), suggesting a possibility of overfitting in the training phase. Besides, some competing models (e.g. the logistic regression, NB approach, DA, and SVM) also performs well in terms of the AUC, *accuracy*, Brier score, and G-mean. Due to the greater proportion of majority samples, the value of *precision (neg)* is always greater than that of *precision (pos)* and the *specificity* greater than the *sensitivity*. On the other hand, the k-means model performs the worst in terms of all indicators, indicating that a lazy learning model might not suit for the context of credit scoring.

In the testing phase, the DA model performs best in terms of the AUC, *precision (neg)*, *sensitivity* and Brier score. The logistic regression model also performs well and scores the highest value in terms of *accuracy* and G-mean, while the KNN model performs the best in terms of *precision (pos)* and *specificity*. Apart from the K-means model, the RF model performs the worst during the testing phase, which indicates that the “nearly perfect” performance of RF model during the training phase is a sign of overfitting. For the Australian dataset, in the training phase, RF model still performs the best among all the competing models in terms of all the indicators except *precision (pos)* and *specificity*. Other models also score high in terms of the AUC and *accuracy*. Furthermore, the logistic regression, DT, and DA present balanced ability of distinguishing the majority and minority classes. Specifically, their scores for *sensitivity* and *specificity* are quite close and the gap between *precision (pos)* and *precision (neg)* remains less than 0.07. As to the other models, the identification is imbalanced; i.e. they are better to identify a certain group of samples. In the testing phase, the DA model performs the best in terms of *accuracy*, *precision (neg)*, *sensitivity* and G-mean, while the logistic model scores the highest in the AUC and lowest in Brier score. At the same time, the KNN model scores the highest *precision (pos)* (0.8373) and the NB model scores the highest *specificity* (0.901). Besides, the SVM and RF model also perform well with *accuracy* greater than 0.85. On the other hand, k-means model performs the worst with most indicators are less than 0.7.

For the Japanese dataset, in the training phase, the RF model and k-means model performs best in terms of all indicators and RF model still achieves the best performance in terms of *accuracy* (0.8594) and *precision (pos)* (0.8287). For the other models, the logistic, DA, and SVM scores a high value of AUC (greater than 0.92) and *accuracy* (greater than 0.86) in the training phase and score the optimal value in terms of the AUC, *precision (pos)*, *specificity*, Brier score and G-mean respectively in the testing phase. Still, K-means model performs the worst with AUC of 0.6409.

For the Taiwan dataset, the RF model performs best in terms of all indicators during the training phase but only in terms of *accuracy* during the testing phase. For the other competing models, the value of AUC remains less than 0.74 in the training phase, declining from the value with small-size datasets. It implies that the identification of the sample structural characteristics becomes more difficult while the sample size grows. As for overall prediction during testing phase, the KNN model scores the highest AUC (0.7213) while the RF model scores the highest *accuracy* (0.6783). For the other competing models, the value of AUC and *accuracy* fluctuates around 0.7 and 0.67 respectively with the k-means model scoring the lowest. As for the identification of minority class, the SVM scores the highest *accuracy* for prediction, with the highest *precision (pos)* (0.7553) followed by the DT model with *precision (pos)* of 0.7415. Meanwhile, the NB model performs the strongest when identifying the bad samples, with the highest *sensitivity* (0.8453). Besides, the NB and SVM model performs best in predicting and identifying the majority class, respectively.

For the HELOC dataset, the RF model performs best in all aspects during the training phase but in no aspects during the testing phase. Specifically, in the training phase, the AUC and *accuracy* remain above 0.7 for all models except the k-means. As for the balance between two groups of samples, the gap between *precision (pos)* and *precision (neg)* remains less than 0.01 for the DT, DA and logistic model. For these three models, the value of Brier score remains less than 0.2 and G-mean greater than 0.71, indicating a better balance ability. In the testing phase, the SVM model performs best in terms of the AUC, *accuracy*, *precision (neg)*, *sensitivity* and G-mean, suggesting excellent generalization. Besides, the NB model performs best in terms of *precision (pos)* and *specificity* while the logistic regression outperforms others in terms of Brier score. In addition, across most of these models, the value of indicators varies within a relatively limited range, which suggests a strong performance of these models with a balanced dataset.

To sum up, the comparison across classical models with different datasets suggests propositions as follows.

First, in the aspect of comprehensive performance, the logistic regression and the DA model achieve a better performance with small-size datasets (e.g. the German dataset, the Australian dataset and the Japanese dataset) during the testing phase while the SVM and NB perform better with large-size datasets (e.g. the Taiwan dataset and the HELOC dataset).

Second, in the aspect of minority class identification (testing phase), the KNN and SVM model achieve (twice, respectively) the best performance in terms of *precision (pos)*, while the NB model performs best with only the HELOC dataset. Besides, the DA model and NB model achieve (twice, respectively) the best performance in terms of *sensitivity*, while the SVM model performs best with only the HELOC dataset. In other word, the SVM and NB perform better when predicting minority class.

Third, in the aspect of balance ability (testing phase), the logistic regression achieves the best performance for three times in terms of Brier score, while the DA and DT model achieve once for each. Besides, the DA and SVM model achieve (twice, respectively) the best performance in terms of G-mean, while the logistic regression model achieves once. By comparison, the DA and the logistic model present better balance in identifying the two groups of samples.

Fourth, in the aspect of robustness (i.e. the performance difference between training and testing phase), the performance during training phase is generally weaker than that during the testing phase. Models on the ground of classical statistics (e.g. the DT and the logistic regression) are more robust than models based on the novel machine learning theories (e.g. the SVM and RF). Besides, after hyper-parameter optimization, the performance difference between the two phases is comparatively small for most baseline models.

Table 6 reports the performance of several state-of-the-art hybrid or ensemble credit-scoring models. These models are constructed in recent literature [29–34] and applied in the context of three prevalent datasets (i.e. the German dataset, the Australian dataset, and the Japanese dataset). We list the value of evaluation metrics as reported in the sourcing literature and present the optimal performance in bold.

**Table 6:** Performance of hybrid or ensemble models in control group.

Source	Framework	Data splitting technique	German dataset					Australian dataset					Japanese dataset				
			*accuracy*	AUC	AUC-H	Brier score	G-mean	*accuracy*	AUC	AUC-H	Brier score	G-mean	*accuracy*	AUC	AUC-H	Brier score	G-mean
Ala’raj and Abbod [30]	ConsA	5-folds	0.7900	0.8020	0.3250	0.1640		0.8810	0.9350	0.6690	0.0960		0.8870	**0.9330**	0.6880	**0.0930**	
Xia, Liu [34]	XGBoost-MS	80%-20%	0.7685		0.2974	0.1655		87.3800		0.6470	0.0908						
	XGBoost-GS	80%-20%	0.7683		0.2977	0.1176		87.8100		0.6557	0.0915						
	XGBoost-RS	80%-20%	0.7718		0.3030	0.0957		87.8200		0.6559	0.0893						
	XGBoost-TPE	80%-20%	0.7734		0.3062	**0.0935**		87.9200		0.6571	**0.0890**						
Tripathi, Edla [29]	NRS+MV	10-folds	0.7798				0.6576	92.3700				0.9226					
	NRS+WV	10-folds	0.7798				0.6576	92.3700				0.9227					
	NRS+LMV	10-folds	0.8298				0.8280	94.9300				0.9480					
	NRS+LWV	10-folds	0.8647				**0.8453**	95.3900				**0.9517**					
Plawiak, Abdar [31]	DGCEC	10-folds						**97.3900**									
Zhang, He [32]	Multi-stage hybrid model	80%-20%	0.7682	0.8029	0.3458	0.1603		0.8754	**0.9370**	**0.7063**	0.0938		0.8720	0.0242	**0.7032**	0.0947	
Xu, Zhang [33]	AGHE	66.6%-33.4%	**0.8760**	**0.8130**	**0.4250**	0.1640		0.8810	0.9250	0.6690	0.0960		**0.8940**	**0.9330**	0.6880	**0.0930**	

Reports the performance of several representative hybrid or ensemble credit-scoring models constructed in recent literature and applied in the context of three prevalent datasets (i.e. the German dataset, the Australian dataset, and the Japanese dataset). The value of evaluation metrics is reported same as the sourcing literature. The bold text indicates the best performance of the row.

As is shown in Table 6, within the context of the three datasets, the performance varies across different models. Within the German dataset, the AGHE presents best overall identification with the optimal values for *accuracy*, AUC, and AUC-H. Meanwhile, the SGBoost-TPE and the NS+LWV score best in terms of Brier score and G-mean, respectively.

Within the Australian dataset, the multi-sage hybrid model scores best in terms of AUC and AUC-H while the DGCEC scores the highest value for AUC. Still, the SGBoost-TPE and the NS+LWV score best in terms of Brier score and G-mean, respectively, as they do within the German dataset.

Three models are included in the comparison within the Japanese dataset. The AGHE and the ConsA score the best values for AUC and brier score while the multi-stage hybrid model and the AGHE score the best in terms of AUC-H and *accuracy*, respectively.

To sum up, across the three datasets (i.e. the German, Australian, and Japanese datasets), the AGHE scores best in terms of the six metrics for overall performance (i.e. the AUC, *accuracy*, *precision (pos)*, *precision (neg)*, *sensitivity*, and *specificity*) and presents strong ability of overall prediction. On the other hand, the XGBoost-TPE and the NRS+LWV presents good balance between the identification of two groups of samples. Compared with the classical models in Table 5, hybrid models in Table 6 performs better in terms of overall prediction and the balanced identification.

### BP-ANN model with increasing hidden layers

The number of hidden layers partly determines the performance of BP-ANN models. However, if we set the number of hidden layers as one of the parameters to optimize, the swarm intelligence algorithms will not work due to the ambiguous dimension of solution space. In this subsection, for instead, we compare the performance of BP-ANN models with different numbers of hidden layers and analyze the robustness of model performance thereby.

We rerun the experiment described in the section of methodology with two and three hidden layers (the number of neurons in the added hidden layers in line with the setting in Table 3) and present the performance in Table 7.

**Table 7:** Performance of BP-ANN models with increasing hidden layers.

**Panel A: BP-ANN models with two hidden layers**									
Dataset	Phase	Indicator	*BA*	*CSO*	*FA*	*GS*	*GWO*	*PSO*	*WSA*
German	Training	*AUC*	0.8859	**0.8989**	0.7257	0.8113	0.6189	0.8535	0.8109
		*accuracy*	0.8313	**0.8398**	0.7190	0.7713	0.7285	0.8050	0.7738
		*precision (pos)*	**0.6115**	0.5844	0.3909	0.4370	0.1785	0.5510	0.4626
		*precision (neg)*	0.9253	0.9488	0.8612	0.9146	**0.9652**	0.9140	0.9053
		*sensitivity*	0.7784	**0.8347**	0.3629	0.6940	0.2732	0.7348	0.6822
		*specificity*	**0.8480**	0.8427	0.7871	0.7918	0.7374	0.8263	0.7998
		Brier score	0.1688	**0.1603**	0.2810	0.2288	0.2715	0.1950	0.2263
		G-mean	**0.7513**	0.7432	0.4127	0.6290	0.2546	0.7089	0.6404
	Testing	*AUC*	0.7898	**0.8001**	0.6939	0.7705	0.6052	0.7888	0.7688
		*accuracy*	**0.7640**	0.7530	0.7070	0.7490	0.7130	0.7610	0.7550
		*precision (pos)*	**0.5071**	0.4342	0.3670	0.3977	0.1177	0.4747	0.4364
		*precision (neg)*	0.8741	0.8938	0.8471	0.8978	**0.9651**	0.8832	0.8994
		*sensitivity*	0.6328	0.6367	0.3425	0.6266	0.2435	0.6377	**0.6442**
		*specificity*	**0.8054**	0.7853	0.7805	0.7775	0.7211	0.7983	0.7863
		Brier score	0.2360	0.2470	**0.2930**	0.2510	0.2870	0.2390	0.2450
		G-mean	0.6647	0.6200	0.3901	0.5915	**0.2073**	0.6438	0.6175
Australian	Training	*AUC*	0.9382	0.9454	0.9395	0.9471	**0.9526**	0.9523	0.9349
		*accuracy*	0.8641	0.8725	0.8743	0.8797	0.8815	**0.8844**	0.8678
		*precision (pos)*	**0.9185**	0.9049	0.9046	0.8982	0.9087	0.8941	0.8996
		*precision (neg)*	0.8205	0.8468	0.8497	0.8649	0.8595	**0.8767**	0.8416
		*sensitivity*	0.8039	0.8261	0.8291	0.8433	0.8398	**0.8532**	0.8214
		*specificity*	**0.9263**	0.9176	0.9177	0.9140	0.9219	0.9119	0.9138
		Brier score	0.1359	0.1275	0.1257	0.1203	0.1185	**0.1156**	0.1322
		G-mean	0.8681	0.8752	0.8766	0.8811	0.8834	**0.8853**	0.8697
	Testing	*AUC*	0.9337	0.9326	0.9189	0.9321	**0.9382**	0.9311	0.9231
		*accuracy*	**0.8638**	0.8623	0.8522	0.8580	0.8580	0.8594	0.8580
		*precision (pos)*	**0.9108**	0.8950	0.8794	0.8711	0.8786	0.8624	0.9076
		*precision (neg)*	0.8317	0.8344	0.8250	0.8484	0.8420	**0.8552**	0.8166
		*sensitivity*	0.8084	0.8137	0.8048	0.8238	0.8169	**0.8291**	0.8027
		*specificity*	**0.9195**	0.9085	0.8990	0.8945	0.8990	0.8873	0.9139
		Brier score	**0.1362**	0.1377	0.1478	0.1420	0.1420	0.1406	0.1420
		G-mean	**0.8696**	0.8640	0.8507	0.8581	0.8590	0.8582	0.8605
Japanese	Training	*AUC*	0.9385	0.9147	0.9376	**0.9439**	0.9418	0.9425	0.8981
		*accuracy*	0.8714	0.8612	0.8692	**0.8746**	0.8739	0.8739	0.8620
		*precision (pos)*	0.8328	0.8211	0.8305	**0.8393**	0.8385	0.8346	0.8009
		*precision (neg)*	0.9191	0.9114	0.9179	0.9184	0.9174	0.9225	**0.9381**
		*sensitivity*	0.9282	0.9247	0.9265	0.9282	0.9274	0.9313	**0.9419**
		*specificity*	0.8152	0.8064	0.8126	**0.8211**	0.8205	0.8180	0.7908
		Brier score	0.1286	0.1388	0.1308	**0.1254**	0.1261	0.1261	0.1380
		G-mean	0.8748	0.8635	0.8730	**0.8779**	0.8769	0.8772	0.8667
	Testing	*AUC*	0.9220	0.8990	0.9268	0.9289	**0.9340**	0.9239	0.8952
		*accuracy*	0.8507	**0.8565**	0.8522	0.8507	0.8493	**0.8565**	0.8522
		*precision (pos)*	0.8193	0.8128	0.8160	0.8182	**0.8231**	0.8199	0.7928
		*precision (neg)*	0.8965	0.9088	0.9017	0.8931	0.8975	0.9055	**0.9238**
		*sensitivity*	0.9060	0.9201	0.9103	0.9054	0.9041	0.9125	**0.9329**
		*specificity*	0.7989	0.7972	0.7930	0.7975	0.8003	**0.8027**	0.7837
		Brier score	0.1493	**0.1435**	0.1478	0.1493	0.1507	**0.1435**	0.1478
		G-mean	0.8558	0.8584	0.8568	0.8543	0.8572	**0.8607**	0.8548
Taiwan	Training	*AUC*	0.7555	**0.7670**	0.6773	0.7496	0.7534	0.7523	0.7545
		*accuracy*	0.6880	**0.7004**	0.6433	0.6881	0.6759	0.6888	0.6870
		*precision (pos)*	0.6502	0.6105	0.4239	0.5848	**0.6698**	0.5662	0.5929
		*precision (neg)*	0.7256	0.7901	**0.8629**	0.7913	0.6818	0.8114	0.7809
		*sensitivity*	0.7070	0.7453	0.6055	0.7390	0.6923	**0.7521**	0.7379
		*specificity*	0.6761	0.6704	0.6104	0.6564	**0.6799**	0.6523	0.6592
		Brier score	0.3120	**0.2996**	0.3568	0.3119	0.3241	0.3113	0.3130
		G-mean	0.6846	**0.6938**	0.5299	0.6793	0.6659	0.6766	0.6769
	Testing	*AUC*	0.7308	0.7335	0.6734	0.7310	**0.7339**	0.7296	0.7327
		*accuracy*	0.6702	0.6818	0.6445	0.6783	0.6615	**0.6820**	0.6723
		*precision (pos)*	0.6361	0.5974	0.4224	0.5736	**0.6533**	0.5552	0.5830
		*precision (neg)*	0.7058	0.7666	**0.8652**	0.7833	0.6717	0.8083	0.7628
		*sensitivity*	0.6876	0.7196	0.6087	0.7268	0.6815	**0.7468**	0.7194
		*specificity*	0.6604	0.6558	0.6106	0.6474	**0.6637**	0.6447	0.6484
		Brier score	0.3298	0.3182	0.3555	0.3217	0.3385	**0.3180**	0.3277
		G-mean	0.6678	**0.6764**	0.5294	0.6701	0.6515	0.6691	0.6624
HELOC	Training	*AUC*	0.7893	0.7963	**0.7969**	0.7933	0.7937	0.7911	0.7955
		*accuracy*	0.7141	0.7229	**0.7231**	0.7190	0.7168	0.7189	0.7227
		*precision (pos)*	0.7741	0.7736	0.7903	0.7752	0.7473	0.7675	**0.8084**
		*precision (neg)*	0.6484	0.6678	0.6497	0.6574	**0.6830**	0.6660	0.6291
		*sensitivity*	0.7102	0.7183	0.7123	0.7139	**0.7253**	0.7169	0.7044
		*specificity*	0.7318	0.7312	0.7412	0.7324	0.7193	0.7273	**0.7509**
		Brier score	0.2859	0.2771	**0.2769**	0.2810	0.2832	0.2811	0.2773
		G-mean	0.7041	**0.7179**	0.7155	0.7116	0.7097	0.7130	0.7128
	Testing	*AUC*	0.7847	0.7904	**0.7909**	0.7887	0.7886	0.7875	0.7899
		*accuracy*	0.7086	**0.7211**	0.7181	0.7139	0.7151	0.7173	0.7170
		*precision (pos)*	0.7680	0.7700	0.7858	0.7702	0.7492	0.7624	**0.8016**
		*precision (neg)*	0.6442	0.6670	0.6444	0.6535	**0.6797**	0.6673	0.6246
		*sensitivity*	0.7057	0.7169	0.7079	0.7103	**0.7218**	0.7157	0.7002
		*specificity*	0.7232	0.7280	0.7356	0.7260	0.7192	0.7247	**0.7431**
		Brier score	0.2914	**0.2789**	0.2819	0.2861	0.2849	0.2827	0.2830
		G-mean	0.6995	**0.7159**	0.7105	0.7071	0.7095	0.7112	0.7072
**Panel B: BP-ANN models with three hidden layers**									
German	Training	*AUC*	0.8230	0.8537	0.8721	0.7752	**0.8927**	0.6352	0.5916
		*accuracy*	0.7853	0.8095	0.8183	0.7545	**0.8345**	0.7288	0.7150
		*precision (pos)*	0.5018	0.5662	0.5650	0.3238	**0.6005**	0.1499	0.1557
		*precision (neg)*	0.9060	0.9136	0.9264	0.9398	0.9352	**0.9776**	0.9537
		*sensitivity*	0.6961	0.7440	0.7718	0.5567	**0.8047**	0.2977	0.2303
		*specificity*	0.8107	0.8318	0.8336	0.7682	**0.8465**	0.7313	0.7319
		Brier score	0.2148	0.1905	0.1818	0.2455	**0.1655**	0.2713	0.2850
		G-mean	0.6714	0.7172	0.7213	0.4826	**0.7464**	0.2377	0.2205
	Testing	*AUC*	0.7764	0.7842	0.7743	0.7400	**0.8047**	0.6155	0.6032
		*accuracy*	0.7460	0.7590	0.7570	0.7430	**0.7690**	0.7290	0.7040
		*precision (pos)*	0.4276	0.4761	0.4470	0.2810	**0.4862**	0.1664	0.1427
		*precision (neg)*	0.8868	0.8819	0.8931	0.9385	0.8899	**0.9665**	0.9482
		*sensitivity*	0.6195	0.6335	0.6397	0.5285	**0.6665**	0.2788	0.1924
		*specificity*	0.7826	0.7971	0.7898	0.7567	**0.8050**	0.7351	0.7270
		Brier score	0.2540	0.2410	0.2430	0.2570	**0.2310**	0.2710	0.2960
		G-mean	0.6115	0.6451	0.6264	0.4479	**0.6491**	0.2468	0.1993
Australian	Training	*AUC*	0.9472	0.9486	0.9269	0.9505	**0.9524**	0.9305	0.9502
		*accuracy*	0.8750	0.8786	0.8562	0.8772	**0.8851**	0.8649	0.8812
		*precision (pos)*	**0.8998**	0.8869	0.8899	0.8738	0.8833	0.8747	0.8910
		*precision (neg)*	0.8549	0.8721	0.8289	0.8799	**0.8864**	0.8572	0.8737
		*sensitivity*	0.8337	0.8497	0.8097	0.8550	**0.8621**	0.8312	0.8498
		*specificity*	**0.9142**	0.9066	0.9086	0.8973	0.9050	0.8961	0.9092
		Brier score	0.1250	0.1214	0.1438	0.1228	**0.1149**	0.1351	0.1188
		G-mean	0.8769	0.8789	0.8570	0.8765	**0.8847**	0.8655	0.8821
	Testing	*AUC*	0.9249	**0.9380**	0.9175	0.9266	0.9300	0.9270	0.9299
		*accuracy*	0.8609	0.8594	0.8493	0.8536	0.8594	0.8464	**0.8681**
		*precision (pos)*	**0.8882**	0.8732	0.8863	0.8401	0.8561	0.8585	0.8711
		*precision (neg)*	0.8371	0.8487	0.8227	**0.8640**	0.8635	0.8345	0.8608
		*sensitivity*	0.8157	0.8235	0.8098	0.8337	0.8306	0.8077	**0.8346**
		*specificity*	**0.9070**	0.8958	0.9015	0.8728	0.8829	0.8830	0.9005
		Brier score	0.1391	0.1406	0.1507	0.1464	0.1406	0.1536	**0.1319**
		G-mean	0.8609	0.8592	0.8501	0.8511	0.8596	0.8455	**0.8648**
Japanese	Training	*AUC*	0.9417	0.9417	0.9412	0.9380	**0.9432**	0.9357	0.9415
		*accuracy*	0.8728	0.8728	0.8714	0.8728	**0.8743**	0.8678	0.8739
		*precision (pos)*	**0.8415**	0.8294	0.8271	0.8296	0.8365	0.8347	0.8366
		*precision (neg)*	0.9117	0.9263	**0.9268**	0.9267	0.9220	0.9084	0.9201
		*sensitivity*	0.9229	**0.9346**	0.9337	0.9341	0.9311	0.9200	0.9292
		*specificity*	**0.8217**	0.8138	0.8110	0.8136	0.8194	0.8153	0.8192
		Brier score	0.1272	0.1272	0.1286	0.1272	**0.1257**	0.1322	0.1261
		G-mean	0.8758	0.8763	0.8755	0.8767	**0.8779**	0.8706	0.8773
	Testing	*AUC*	0.9201	0.9156	0.9231	**0.9333**	0.9156	0.9269	0.9259
		*accuracy*	0.8507	0.8522	0.8536	**0.8580**	0.8464	0.8522	0.8493
		*precision (pos)*	**0.8266**	0.8248	0.8120	0.8224	0.8188	0.8238	0.8190
		*precision (neg)*	0.8836	0.8905	**0.9057**	0.9023	0.8802	0.8892	0.8868
		*sensitivity*	0.8998	0.9074	**0.9141**	0.9119	0.8977	0.9025	0.9009
		*specificity*	0.7972	**0.8025**	0.7946	0.8011	0.7925	0.7995	0.7983
		Brier score	0.1493	0.1478	0.1464	**0.1420**	0.1536	0.1478	0.1507
		G-mean	0.8543	0.8552	0.8571	**0.8615**	0.8488	0.8557	0.8513
Taiwan	Training	*AUC*	0.7608	0.7594	0.7245	0.6683	0.7532	**0.7695**	0.7565
		*accuracy*	0.6937	0.6868	0.6675	0.6298	0.6923	**0.6993**	0.6838
		*precision (pos)*	0.6256	**0.6523**	0.5619	0.6349	0.5742	0.5640	0.5966
		*precision (neg)*	0.7620	0.7214	0.7734	0.6273	0.8105	**0.8343**	0.7718
		*sensitivity*	0.7253	0.7052	0.7267	0.6785	0.7544	**0.7783**	0.7423
		*specificity*	0.6709	**0.6763**	0.6398	0.5057	0.6559	0.6585	0.6611
		Brier score	0.3063	0.3132	0.3325	0.3702	0.3077	**0.3007**	0.3163
		G-mean	**0.6898**	0.6832	0.6533	0.5195	0.6812	0.6834	0.6681
	Testing	*AUC*	0.7340	**0.7360**	0.7169	0.6609	0.7356	0.7339	0.7321
		*accuracy*	0.6758	0.6723	0.6640	0.6373	0.6785	**0.6798**	0.6735
		*precision (pos)*	0.6097	0.6341	0.5570	**0.6376**	0.5580	0.5428	0.5863
		*precision (neg)*	0.7418	0.7101	0.7698	0.6270	0.7996	**0.8178**	0.7576
		*sensitivity*	0.7030	0.6900	0.7220	0.6808	0.7367	**0.7511**	0.7288
		*specificity*	0.6554	**0.6610**	0.6375	0.5104	0.6440	0.6423	0.6501
		Brier score	0.3242	0.3277	0.3360	0.3627	0.3215	**0.3202**	0.3265
		G-mean	**0.6721**	0.6687	0.6476	0.5222	0.6675	0.6645	0.6541
HELOC	Training	*AUC*	0.7969	**0.7985**	0.7960	0.7894	0.7976	0.7348	0.7982
		*accuracy*	0.7202	**0.7251**	0.7203	0.7165	0.7240	0.6806	0.7224
		*precision (pos)*	0.8127	0.7963	0.8124	0.7394	0.7545	**0.8441**	0.7436
		*precision (neg)*	0.6193	0.6473	0.6197	0.6919	0.6906	0.5023	**0.6991**
		*sensitivity*	0.7013	0.7124	0.7013	0.7256	0.7274	0.6672	**0.7310**
		*specificity*	**0.7545**	0.7465	0.7541	0.7122	0.7210	0.5988	0.7164
		Brier score	0.2798	**0.2749**	0.2797	0.2835	0.2760	0.3194	0.2776
		G-mean	0.7077	0.7169	0.7080	0.7131	**0.7214**	0.5679	0.7197
	Testing	*AUC*	0.7905	0.7906	0.7898	0.7854	**0.7909**	0.7346	0.7897
		*accuracy*	0.7149	**0.7171**	0.7157	0.7153	0.7169	0.6821	0.7165
		*precision (pos)*	0.8053	0.7879	0.8079	0.7373	0.7475	**0.8444**	0.7381
		*precision (neg)*	0.6167	0.6400	0.6151	0.6898	0.6837	0.5044	**0.6928**
		*sensitivity*	0.6982	0.7052	0.6976	0.7246	0.7210	0.6682	**0.7266**
		*specificity*	0.7461	0.7355	**0.7475**	0.7091	0.7137	0.6003	0.7097
		Brier score	0.2851	**0.2829**	0.2843	0.2847	0.2831	0.3179	0.2835
		G-mean	0.7026	0.7095	0.7034	0.7108	**0.7143**	0.5693	0.7133

Reports the performance of BP-ANN models with the three datasets (i.e. the German, Australian, Japanese, Taiwan, and HELOC dataset). Parameters are trained by seven different swarm algorithms (i.e. BA, CSO, FA, GS, GWO, PSO, and WSA). Eight indicators (i.e. AUC, *accuracy*, *precision (pos)*, *precision (neg)*, *sensitivity*, *specificity*, Brier score, and G-mean) are reported as metrics for performance evaluation. The bold text indicates the best performance of the row. Panel A reports the performance of BP-ANN model with two hidden layers while Panel B reports the performance of BP-ANN model with three hidden layers.

Comparing the results in Table 7 with those in Table 6, we notice that, despite the fluctuating scores in terms of the 8 indicators, the fitness of training as well as the generalization of test are improved with the increased number of hidden layers. To be specific, while the number of hidden layers goes from one to two and to three, for the German dataset the optimal AUC moves from 0.8859 to 0.8989 and to 0.8927 in the training phase and from 0.8004 to 0.8001 and to 0.8047 in the testing phase; for the Australian dataset, it moves from 0.9514 to 0.9526 and to 0.9524 in the training phase and from 0.9373 to 0.9382 and to 0.9380 in the testing phase; for the Japanese dataset, it moves from 0.9455 to 0.9439 and to 0.9432 in the training phase and from 0.9354 to 0.9340 and to 0.9333 in the testing phase; for the Taiwan dataset, it moves from 0.7596 to 0.767 and to 0.7695 in the training phase and from 0.7403 to 0.7969 and to 0.7985; for the HELOC dataset, it moves from 0.7936 to 0.767 and to 0.7695 in the training phase and from 0.7898 to 0.7909 and to 0.7909 in the testing phase. The trend of AUC indicates that BP-ANN with more hidden layers outperform those with less hidden layers and such outperformance is evident when comparing the scores of other indicators.

We conduct further comparison with models in the control group (see Tables 5 and 6) and find that BP-ANN performs better with increased number of hidden layers. In the context of the German dataset, the model of Zhang, He [32] and that of Xu, Zhang [33] in the control group outperform our BP-ANN model with two hidden layers. However, in the context of other datasets, our optimized BP-ANN model with increased hidden layers outperforms any model in the control group.

Therefore, we propose that the fitness and generalization of BP-ANN models improve with the number of hidden layers increasing. Notably, the model performs comparatively stably (with fluctuation within an acceptable extent) while the number of hidden layers increasing, which indicates greater robustness of BP-ANN trained by SI algorithms. In addition, increasing hidden layers would not lead to overfitting. Thus, we recommend that users train BP-ANN models with different number of hidden layer in order to find out the optimal setting for the certain context.

### Time complexity

When selecting the applicable model to score credit, we take the complexity as well as accuracy into consideration. Table 8 reports the time complexity of BP-ANN models trained by different swarm intelligence algorithms. Each algorithm contains 10 individuals and iterates 20 times on the same computer.

**Table 8:** Time complexity of BP-ANN models trained by swarm intelligence algorithms.

Swarm intelligence algorithm	German dataset	Australian dataset	Japanese dataset	Taiwan dataset	HELOC dataset
*BA*	67.671	61.114	56.414	307.952	383.518
*CSO*	73.088	64.143	54.089	413.748	502.731
*FA*	70.010	57.387	87.640	340.765	665.824
*GS*	50.981	32.897	30.548	139.787	267.296
*GWO*	79.411	42.679	37.937	186.706	340.226
*PSO*	36.032	43.832	22.776	338.315	667.629
*WSA*	40.729	26.095	25.772	133.581	200.251

Reports the time complexity of BP-ANN models with the five datasets (i.e. the German, Australian, Japanese, Taiwan, and HELOC dataset). Parameters are trained by seven different swarm algorithms (i.e. BA, CSO, FA, GS, GWO, PSO, and WSA). The numbers in indicates how many seconds the model runs.

As shown in Table 8, the time complexity varies across different datasets with uniform parameter set. Within the context of small-size dataset (e.g. the German, Australian and Japanese dataset), the PSO and the WSA optimize the most efficiently (i.e. runs within 50 sec) while the CSO and the FA optimize with least efficiency (more than 1 min). For the other SI algorithms, the optimization consumes around one minute. When the dataset is small sized, the time complexity varies within one minute.

However, within the large-size datasets, the time complexity increases while the size of dataset grow. Within the Taiwan dataset, the WSA consumes least time (133.581 sec) followed by the GS (2 min) and the GWO (3 min) while the CSO consumes the most (nearly 7 min). Within the HELOC dataset, despite longer running time because of the increased sample size, the WSA consumes still the least time (approx. 3.5 min) followed by the GS (267.296 sec) and the GWO (340.226 sec) while the FA and the PSO consumes the most (approx. 11 min). In other word, the WSA, GS, and GWO consume less time and perform more robustly with large-size datasets.

### Analytic comparison

This section is intended to answer whether our BP-ANN model trained by SI algorithms outperforms the classical and state-of-the-art models for credit scoring.

#### First, the overall prediction

Within the German dataset, the PSO-BP-ANN (with the AUC of 0.8004) outperforms most models in the control group, albeit slightly weaker than that of [30, 32, 33]. Within the other four datasets, our model outperforms all the models in the control group. Specifically, the optimal AUC measures 0.9370 for the control group but 0.9373 for our model within the Australian dataset; 0.9330 for the control group but 0.9354 for our model within the Japanese dataset; 0.7213 for the control group but 0.7403 for our model within the Taiwan dataset; and 0.7851 for the control group but 0.7898 for our model within the HELOC dataset. In addition, our model presents best performance in terms of the mean value.

#### Second, balanced prediction during testing phase

Unlike the BP-ANN whose output is the predicted probability of credit default, models with output in the form of labels (e.g. the DT, RF, and SVM) present outstanding performance in binary classification. With a uniform threshold of classification (0.5), these models outperform ours in terms of balance metrics (i.e. *accuracy*, Brier score, and G-mean). Nevertheless, our model performs better than the numerical regression models in the control group.

#### Third, robustness of prediction

We focus on the value range of evaluation metrics for each model. Some state-of-the-art models are excluded from this comparison, for the evaluation metrics are missing during training phase. Within the German dataset, the average range for control group is 0.4313 (training) and 0.185 (test), while the average range for our models is 0.1008 (training) and 0.0608 (test). Within the Australian dataset, the average range for control group is 0.4089 (training) and 0.2552 (test), while the average range for our models is 0.0203 (training) and 0.0195 (test). Within the Japanese dataset, the average range for control group is 0.4242 (training) and 0.5148 (test), while the average range for our models is 0.0091 (training) and 0.0145 (test). Within the Taiwan dataset, the average range for control group is 0.5148 (training) and 0.2323 (test), while the average range for our models is 0.0687 (training) and 0.0656 (test). Within the HELOC dataset, the average range for control group is 0.3675 (training) and 0.1400 (test), while the average range for our models is 0.0274 (training) and 0.0282 (test). Thus, with less variant prediction, our model performs with increasing robustness across datasets.

#### Fourth, time complexity

Baseline models in the control group conduct grid search to determine the hyper-parameter set. Consequently, their time complexity is several times of ours. As is proposed in prior literature [26–28], some state-of-the-art techniques to determine neural network architecture requires several GPU-days. However, with small-size datasets, the hyper-parameters of BP-ANN are determined by SI algorithms within one minute (i.e. 59.703 sec, 46.878 sec, and 45.025 sec for the German, Australian, and Japanese dataset, respectively). With large-size dataset, the process completes within 12 min (i.e. 265.836 sec and 432.497 sec for the Taiwan and HELOC dataset, respectively). Instead of grid search for the optimal parameter set, our models conducts guided search based on available information. Therefore, our models consume acceptable runtime to determine parameter set for the BP-ANN with comparatively good performance, which is more practical in real world.

To sum up, the prominent advantage of our framework lies in that it searches the hyper-parameter space of BP-ANN within acceptable runtime and determines a preferable hyper-parameter set efficiently. The fitness and generalization of BP-ANN are improved thereby. In addition, our models predict more precisely when the new samples are “bad”. Furthermore, our models enjoy greater robustness while the performance varies limitedly between training and testing phase.

## Conclusions

This paper proposes a novel framework for credit scoring which is conducted in three steps. First, pre-processing of data, including imputation to make up the missing values, normalization to eliminate the effect of measurement, and re-ordering to balance the occurrence of sample with binary labels. Second, training the model. We employ several SI algorithms to optimize the hyper-parameters of BP-ANN and determine the optimal algorithm based on the value of AUC. The search space of hyper-parameters is set in line with prior literature. Third, applying the model. We apply the optimal model determined in the second step to predict new samples pre-processed in the first step.

Our framework determines a preferable hyper-parameter set for the BP-ANN with acceptable runtime and thereby improves the fitness and generalization of neural networks. By comparison with classical and hybrid or ensemble models in the control group, our framework performs more robustly across training and testing phases. Additionally, models proposed in this paper predict with greater precision when applied to credit default samples.

An interesting follow-up idea is to develop an ensemble or hybrid version of SI-training BP-ANN. In order to improve the identification of minority class, we recommend that the penalty factor for identification error of minority class be included as hyper-parameter to train the neural network. Furthermore, a body of evaluation metrics (e.g. *precision (pos)*, Brier score, G-mean, etc.) are employed and thereby hyper-parameters of BP-ANN are optimized with multiple objectives.
